# The lactate metabolism and protein lactylation in epilepsy

**DOI:** 10.3389/fncel.2024.1464169

**Published:** 2025-01-14

**Authors:** Xi Kuang, Shuang Chen, Qingmei Ye

**Affiliations:** ^1^Hainan Health Vocational College, Haikou, China; ^2^Department of Neurology, Hubei Provincial Hospital of Integrated Chinese and Western Medicine, Hubei University of Chinese Medicine, Wuhan, China; ^3^Hainan General Hospital and Hainan Affiliated Hospital of Hainan Medical University, Haikou, China

**Keywords:** protein lactylation, histone deacetylase, epilepsy, high-mobility group box 1, hypoxia-inducible factor-1α, lactate

## Abstract

Protein lactylation is a new form of post-translational modification that has recently been proposed. Lactoyl groups, derived mainly from the glycolytic product lactate, have been linked to protein lactylation in brain tissue, which has been shown to correlate with increased neuronal excitability. Ischemic stroke may promote neuronal glycolysis, leading to lactate accumulation in brain tissue. This accumulation of lactate accumulation may heighten neuronal excitability by upregulating protein lactylation levels, potentially triggering post-stroke epilepsy. Although current clinical treatments for seizures have advanced significantly, approximately 30% of patients with epilepsy remain unresponsive to medication, and the prevalence of epilepsy continues to rise. This study explores the mechanisms of epilepsy-associated neuronal death mediated by lactate metabolism and protein lactylation. This study also examines the potential for histone deacetylase inhibitors to alleviate seizures by modifying lactylation levels, thereby offering fresh perspectives for future research into the pathogenesis and clinical treatment of epilepsy.

## Highlights

- The lactate metabolism and transport carried out in neurons and glial cells may influence the development of epilepsy by regulating protein lactylation levels.
- Lactate may mediate epilepsy-related neuronal loss by promoting HMGB1 lactylation.
- Ischemic stroke may promote HMGB1 lactylation by activating HIF-1, which can promote a shift in the mode of cellular energy acquisition from oxidative phosphorylation to glycolysis, thereby inducing post-stroke epilepsy.
- Histone deacetylases may affect protein lactylation by regulating the transcriptional activity of HIF-1.
- Histone deacetylase inhibitors may combat post-stroke epilepsy by modulating hypoxia-induced protein lactylation.

## 1 Introduction

Epilepsy, as a common chronic disease of the nervous system, has affected more than 70 million people of all ages worldwide, with the number continuing to rise annually (Eugen et al., 2019). The factors inducing seizures are diverse and multifaceted. Ischemic stroke is one of the common causes of epilepsy. Post-ischemic stroke epilepsy accounts for approximately 9% of all epilepsy cases, a figure slightly lower than that caused by cerebral hemorrhage. However, the growing number of individuals affected by ischemic stroke each year highlights its significant impact on public health (Lanqing et al., 2021; Carolina et al., 2021). The hypoxic environment caused by ischemic stroke often forces brain tissue to obtain energy through glycolysis, thereby producing a large amount of lactate.

Lactate in the brain may affect the progression of various neuropsychiatric diseases through its roles in learning, memory, and emotional regulation. This process may be related to lactate-mediated post-translational modifications of proteins (Hagihara et al., 2021). Lactylation (La), a novel type of post-translational protein modification, was identified by Zhang et al. (2019). With the ongoing research into protein lactylation modification in recent years, HAGIHARA H et al. found that lysine lactylation (Kla) may be prevalent in neurons and glial cells in the brain.

Furthermore, the level of lysine lactylation is regulated by lactic acid levels, neural excitation, and social defeat stress (Hagihara et al., 2021). It suggests that increased neuronal excitability may be closely related to the level of protein lactylation in neurons.

Abnormal excitation of neurons is a key driver of epileptogenesis. Ischemia/reperfusion injury (I/R) can induce epileptogenesis by mediating abnormal excitation of neurons (Paudel et al., 2020; Esih et al., 2021; Endres et al., 2022). It can be speculated that ischemia-induced stroke may lead to increased glucose metabolism in the brain, resulting in the production of large quantities of lactate, which can be transformed into acetyl groups to participate in the acetylation of proteins related to neuronal excitation and thus cause post-stroke epilepsy (Hagihara et al., 2021; He et al., 2023). That is, protein lactylation may be a potential regulatory target for post-ischemic stroke epilepsy. While anti-epileptic drugs can help most patients control their seizures, approximately 30% of individuals with epilepsy do not respond to current clinical medications (Sun et al., 2021). This review mainly focuses on the impact of lactate metabolism and protein acetylation on epilepsy and changes in acetylation modifications controlled by histone acetyltransferases (HATs) and histone deacetylases (HDACs). Furthermore, based on the mechanisms of action of HDAC inhibitors (HDACIs) in regulating acetylation modifications, we discuss the possibility that they could provide a new strategy for treating post-stroke epilepsy.

## 2 The lactate mediates epilepsy by regulating the functions of neurons and glial cells

Seizures are usually localized in the hippocampus, with temporal lobe epilepsy (TLE) being the most common (Sun et al., 2021). Previous studies have suggested that neuronal hyperexcitability in epilepsy is primarily mediated by neuronal death and neuroinflammation, which is mediated by astrogliosis and microglial activation, and the relationship between neuronal death and neuroinflammation may be complex (Haenisch et al., 2015; Roh et al., 2023). Seizures are usually accompanied by significant glial cell proliferation and loss of neurons, and glial cell proliferation-mediated neuroinflammation often leads to neuronal death (Feng et al., 2019). Protein lactylation modifications have been found in the hippocampus and prefrontal cortex and are strongly associated with neuronal excitability (Hagihara et al., 2021).

Currently, there is insufficient evidence to confirm that protein lactylation directly induces epilepsy by regulating neuronal excitability. However, lactate, as the donor of lactide, can serve as an energy source for metabolic activities in neurons and glial cells during ischemia and hypoxia, potentially influencing the progression of post-stroke epilepsy.

### 2.1 Glial cells mediate epilepsy by regulating the release of neurotransmitters

Studies on status epilepticus suggest that astrocyte and microglial activity changes, which influence critical homeostatic processes—such as synaptogenesis, extracellular ion concentrations, and excitation-inhibition balance—may occur early in epilepsy development. Inhibiting glial cell activity has been shown to reduce susceptibility to epilepsy (Shen et al., 2023). Neuroglia, such as astrocytes, microglia, and oligodendrocytes, may influence neuronal excitability by regulating the production of glutamate, adenosine triphosphate (ATP), and γ-aminobutyric acid (GABA) in the central nervous system, thereby modulating the progression of epilepsy (Shen et al., 2023).

Glutamate is the primary excitatory neurotransmitter in the central nervous system and can induce seizures by increasing synaptic transmission in the hippocampal region. Although the death of neurons caused by cerebral infarction can reduce the synthesis of the neurotransmitter glutamate, previous studies have confirmed that the level of glutamate in the blood and cerebrospinal fluid of ischemic stroke patients is related to the severity of infarction and neurological dysfunction (Nicolo et al., 2019). Research has shown that stroke can mediate neuroinflammatory damage by activating astrocytes’ TGF-β pathway, and the TGF-β signaling in astrocytes may have a pro-epileptic effect (Korotkov et al., 2020). Therefore, astrocytes may induce post-stroke epilepsy by mediating stroke-related glutamate excitotoxicity. Astrocytes primarily take up excessive glutamate from the synaptic cleft through two types of glutamate transporters, such as glutamate–aspartate transporter (GLAST) and glutamate transporter-1 (GLT1). The glutamate, which is transported into the cell by GLAST or GLT1, can be converted into glutamine by glutamine synthetase, which is a major precursor for the biosynthesis of the inhibitory neurotransmitter GABA (Sun et al., 2021; Peterson et al., 2021). Glutamate released by neurons is taken up by astrocytes and converted to glutamine to alleviate the excitotoxicity of glutamate on neurons. Glutamine serves as a source of energy for both glial cells and neurons (Nagy et al., 2018). Recent studies have shown that reducing the expression of nascent proteins and the activity of inward rectifying K+ channel subtype 4.1 (Kir4.1), respectively, leads to a decrease in the expression of GLAST and GLT1 in hippocampal astrocytes, which results in increased extracellular glutamate levels and decreased GABA release, leading to an increased risk of epileptogenesis (Sun et al., 2021; Centonze et al., 2023). Since knocking down nascent protein does not affect the expression of GABA transporter proteins in astrocytes, the reduction in GABA release may be attributed to reduced GABA synthesis (Sun et al., 2021). It has been shown that inhibiting the conversion of putrescine to spermine in astrocytes would lead to more remaining putrescine being available for the synthesis of GABA, thereby effectively reducing epileptic activity (Kovács et al., 2021).

Previous research suggests that compared to GLAST, GLT1 is the main transporter for the uptake of glutamate by astrocytes (Peterson et al., 2021). In the intrahippocampal kainic acid model of TLE, GLT1 regulates the frequency of seizures and the total time spent in seizures by mediating the uptake of most glutamate in the dorsal forebrain (Peterson et al., 2021). GLT1 mitigates ischemic stroke-induced neuroexcitotoxicity by increasing glutamate reuptake in astrocytes, thereby reducing neuronal cell death, which is an important cause of post-stroke epilepsy (Wang et al., 2022). Post-translational modifications of GLT-1, including palmitoylation, ubiquitination, nitrosylation, and succinylation, can regulate the distribution of GLT-1 and the rate at which it transports glutamate (Peterson et al., 2021). Although it has not yet been confirmed that palmitoylation can alter the activity of GLT1, based on reports linking protein palmitoylation to neuronal excitability, GLT1 lactylation may affect the excitability of neurons by regulating the concentration of glutamate between synapses (Hagihara et al., 2021).

Previous studies have shown that calcium ions mediate the release of glutamate and ATP from neurotransmitter-activated astrocytes to promote neuronal firing patterns (Shen et al., 2023; Chen et al., 2023). However, other research suggests that the majority of the neurotransmitter glutamate in the brain is synthesized and released by neuronal cells, with astrocytes playing a role in regulating the concentration of glutamate between synapses through reuptake. Until the concept of “glutamatergic astrocytes” was proposed by DeCeglia et al., the ability of astrocytes to transmit information like neurons was unknown. Glutamatergic astrocytes are located mainly in the hippocampus and express the vesicular glutamate transporter protein 1 (vGLUT1), which specifically loads glutamate into synaptic vesicles and promotes its release into the synaptic gap (de Ceglia et al., 2023). Although vGLUT1-mediated glutamate release may promote the occurrence of epilepsy, it does not prove that the glutamate transported by vGLUT1 is synthesized by astrocytes rather than acquired by astrocytes from the synaptic cleft. Glutamate can not only directly induce epilepsy by mediating the excitotoxicity of neurons but also induce epilepsy related to brain tissue damage by altering the function of inhibitory interneurons. Previous studies have confirmed that brain tissue damage can lead to an increase in synaptic inputs generated by pyramidal neurons, thereby driving an increase in the excitability of surviving hilar interneurons, which in turn leads to the occurrence of post-traumatic epilepsy (Butler et al., 2017). This may be closely related to the glutamatergic synapses formed between pyramidal cells and interneurons in the hippocampus, as a previous study has shown that pyramidal cells may regulate the transition between inhibitory interneurons and disinhibitory interneurons through the glutamatergic synapses formed with interneurons (Tzilivaki et al., 2023).

During status epilepticus, neurons, astrocytes, and microglia release ATP, activating the purinergic receptor P2Y1 on astrocytes and further exacerbating neuronal excitotoxicity. Meanwhile, ATP released from damaged neurons can activate NLRP3 inflammatory vesicles by binding to the ATP-gated ion channel P2X7 in microglia, mediating inflammatory injury in brain tissue (Liu et al., 2022). Glial cells and neurons can sustainably increase neuronal excitotoxicity by releasing excitatory neurotransmitters. However, adenosine kinase synthesized by astrocytes converts ATP to adenosine, alleviating neuronal excitotoxicity by activating presynaptic A1 adenosine receptors, thereby blocking this self-sustaining cycle of neuronal activity (Shen et al., 2023; Boison and Steinhäuser, 2018). Meanwhile, as brain-resident immune cells, microglia catalyze the conversion of ATP to adenosine by expressing CD39, an extracellular ATP/ADP hydrolase encoded by Entpd1, which can reduce seizures (Hu et al., 2023). Although activated microglia may promote the occurrence of epilepsy by releasing glutamate and inflammatory cytokines (such as interleukin-1β, interleukin-6, and tumor necrosis factor-α), inhibiting glutamate uptake, and reducing GABAergic transmission (Shen et al., 2023), the above-mentioned astrocytes and microglia can play a dual role in promoting and inhibiting epilepsy, which may depend on different stages of epileptogenesis.

Previous studies have shown that oligodendrocytes, like astrocytes and microglia, regulate the release and reuptake of neurotransmitters between neuronal synapses, thereby mediating epileptogenesis and development. Oligodendrocytes have been shown to be involved in the regulation of glutamate levels in the brain (Shen et al., 2023).

### 2.2 Lactate interference with glial cell function

Glucose is the main energy source for the brain, and interfering with the glucose metabolic process may affect brain function. Lactate produced by glycolysis may mediate glial cell activation and neuronal functional impairment by promoting histone acetylation (Pan et al., 2022; Wei et al., 2023). The increase in histone H3K18 acetylation levels in the hippocampus can directly activate the nuclear factor kappa-B (NF-κB) signaling pathway by stimulating the promoters of Rat transcription factor (Rela) and NF-κB, which can enhance the production and release of pro-inflammatory factors and exacerbate the inflammatory damage associated with epilepsy (Wei et al., 2023; Cai and Lin, 2022). This pathological change may be amplified by a positive feedback loop between glycolysis/H4K12la/ pyruvate kinase isozyme type M2 (PKM2). As a key enzyme in the glycolytic pathway, PKM2 can ensure that the cell’s energy metabolism switches from oxidative phosphorylation to glycolysis. The H3K18la mainly mediates the pro-inflammatory activation of microglia through the NF-κB signaling pathway. Microglia activation can exacerbate neuronal functional damage by promoting the formation of NLRP3 inflammasomes and Apoptosis-Associated Speck-Like Protein Containing a Caspase Recruitment Domain (ASC) specks (Pan et al., 2022).

**Figure 1:**
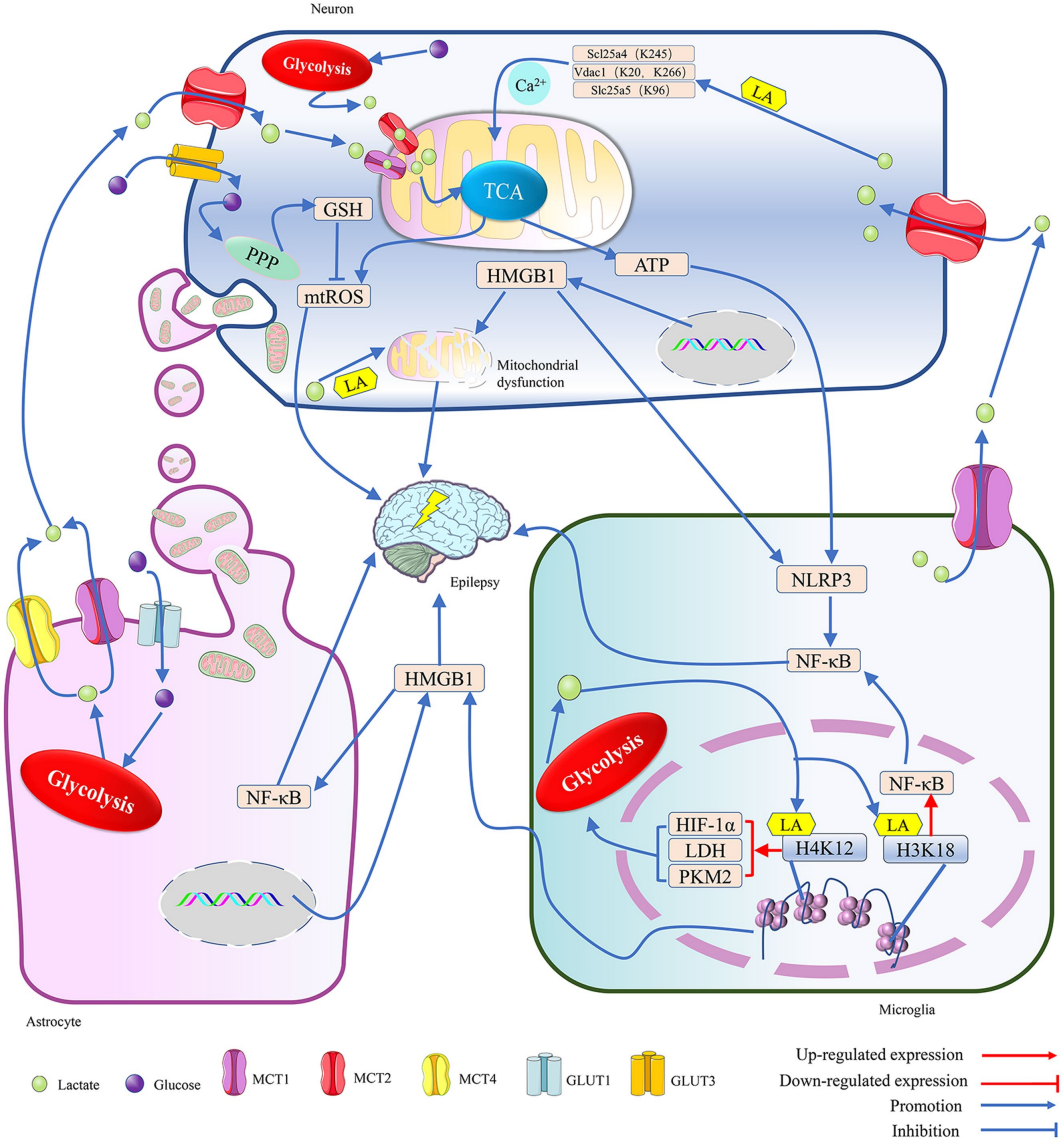
The metabolic crosstalk between neurons and glia involving lactate is implicated in epilepsy. 1. Both glucose and lactate can serve as energy substrates for neurons. Glucose is primarily taken up by neurons via GLUT3, while lactate, mainly provided by astrocytes and microglia, is mostly taken up by neurons via MCT2 during situations of intense stimulation of neural function. In a hypoxic environment, within neurons, the lactate generated from glucose through glycolysis and the lactate transferred into the cell by MCT2 are spread on the mitochondrial membrane by MCT1 and MCT2. There, they penetrate the TCA cycle, and the TCA contributes to the production of a significant amount of ATP and ROS through the promotion of oxidative phosphorylation. The ATP not only supports neuronal excitation but also activates the NLRP3/NF-κB pathway in microglia, leading to epilepsy. The mtROS may cause epilepsy by inducing oxidative stress in neurons, but PPP can reduce it by synthesizing glutathione, an antioxidant. Additionally, lactate can lead to epilepsy by enhancing the lactylation levels of proteins related to mitochondrial function, such as inducing Ca2^+^ overload-mediated mitochondrial apoptosis by promoting lactylation of key proteins of the Ca2^+^ signaling pathway, Scl25a4 (K245), Slc25a5 (K96) and Vdac1 (K20, K266). The transfer of HMGB1 from the nucleus to the cytoplasm can help coordinate homophilin lactylation, leading to epileptogenesis by mediating neuronal mitochondrial dysfunction. However, astrocytes maintain normal neuronal physiological activity and reduce epileptogenesis by providing healthy mitochondria to neurons. 2. In astrocytes, glucose taken up by the GLUT1 channel is converted into lactate, which is primarily exported by the MCT1/4 and used as an energy source by neurons. Both microglia and astrocytes can secrete HMGB1, which is a damage-associated molecular pattern and continues to induce the activation of glial cells and the inflammatory damage of neurons, leading to the occurrence of epilepsy. Meanwhile, HMGB1 secreted by microglia can mediate epileptogenesis through activation of the NF-κB pathway in astrocytes. 3. In a hypoxic environment, the lactate generated by glycolysis in microglia not only supports neuronal energy metabolism but also facilitates the lactylation of histones H3K18 and H4K12, which enhances the expression of NF-κB and glycolysis-related genes. NF-κB can trigger the onset of epilepsy, and the enzymes, such as LDH, HIF-1α, and PKM2, can further promote the production of lactate, which then helps in the lactylation of histones, ultimately intensifying the symptoms of epilepsy. Adenosine triphosphate (ATP); glucose transporter 1/3 (GLUT1/3); glutathione (GSH); high-mobility group box 1 (HMGB1); hypoxia-inducible factor 1 alpha (HIF-1α); K (Lysine); monocarboxylate transporter (MCT); mitochondria reactive oxygen species (mtROS); NLR family pyrin domain containing 3 (NLRP3); nuclear factor kappa-B (NF-κB); pentose phosphate pathway (PPP); pyruvate kinase isozymes M2 (PKM2); and tricarboxylic acid cycle (TCA).

Microglia cells have significantly higher levels of H4K12la at the promoters of hypoxia-inducible factor-1α (HIF-1α), PKM2, and lactate dehydrogenase (LDH), which leads to increased expression of these glycolysis-related proteins and further upregulates H3K18 and H4K12 acetylation (Pan et al., 2022; Wei et al., 2023). While there was no significant difference in H4K12la between the transgenic AD model mice and wild-type mice in the affected areas of their brains, the research suggests that the metabolism of astrocytes and neurons, like microglia, is regulated by lactate (Pan et al., 2022; Barros et al., 2023). Previous studies have suggested that cognitive impairment may be linked to the expression levels and activities of key enzymes in glycolysis, such as LDH, as well as glucose transporter protein 1 (GLUT1) and GLUT3, which are the primary cellular channels for glucose uptake in neurons and astrocytes, respectively (Yang et al., 2024; Wu et al., 2023). It is, therefore, possible that the glucose uptake of astrocytes and neurons may affect brain function by regulating lactate production. Based on the evidence of crosstalk between microglia, astrocytes, and neurons, the functions of starry glial and neuronal cells may also be influenced by protein acetylation, as in microglia (Mayorga-Weber et al., 2022; Mason, 2017). The lactate/H4K12la/PKM2 feedback loop in microglia might continuously promote the acetylation of histones in neurons and astrocytes by providing lactate. Furthermore, as GLUT1 on astrocytes transports more glucose into the cell, aerobic glycolysis gradually replaces mitochondrial oxidative phosphorylation as these cells’ main glucose metabolic pathway. The glucose taken up by neurons via GLUT3 not only participates in oxidative energy supply but also is metabolized by the pentose phosphate pathway to eventually produce glutathione to maintain redox balance (Mayorga-Weber et al., 2022) (Figure 1).

Cerebral ischemia can induce the proliferation of astrocytes and stimulate their glycolytic metabolism (Lv et al., 2015), leading to the release of lactate, which can then enter neuron cells through monocarboxylic acid transporter (MCT) to cause an increase in neuronal activity (Barros et al., 2023; Yang et al., 2024; Wu et al., 2023). With the release of lactate from astrocytes, the negative feedback on glycolysis gradually weakens, allowing astrocytes to continuously produce lactate for energy transport to the neuron cells (Barros et al., 2023). Additionally, it has been demonstrated that neurons can uptake lactate released by microglia via MCT2 (Mayorga-Weber et al., 2022). Consequently, microglia and astrocytes may maintain neuron energy metabolism by continuously supplying lactate. This lactate may affect the physiological functions of neurons by promoting histone lactylation in hippocampal tissue (Nagy et al., 2018; Wei et al., 2023) (Figure 1).

MCT1-4 show cell-type-specific distribution. MCT1/4 is primarily located on the membranes of astrocytes, with MCT4 being unique to astrocytes. MCT2 is expressed exclusively by neurons, and microglia mainly rely on MCT1 for lactate transport (Yang et al., 2024; Jády et al., 2016). Lactate/pyruvate is transported between cells via MCTs. The imported pyruvate may enter the mitochondria and preferentially serve as a substrate for the pyruvate dehydrogenase system (PDHc) to provide energy for the cells, while the imported lactate is first oxidized by LDH to produce NADH, which then follows a specific shuttle mechanism to enter the mitochondria. When the shuttling of NADH is impeded, it may prevent the oxidation of lactate (Nagy et al., 2018) as a product of glycolysis; lactate/pyruvate can be used by the mitochondria for oxidative phosphorylation. Astrocytes can release lactate through MCT1/4, which may then enter neuronal cells via MCT2. Finally, the lactate enters the mitochondria through the MCT1/2 channels in the neuron’s cytoplasm (Yang et al., 2024; Wu et al., 2023; Yang et al., 2022), promoting mitochondrial energy metabolism and reactive oxygen species (ROS) production. The mitochondria ROS (mtROS) can induce dysfunction of neuronal mitochondria and damage the structure and function of synaptic elements (Jia et al., 2021). Moreover, the uptake of dysfunctional mitochondria released from glial cells by neurons can also lead to abnormalities in neural discharge (Zhang et al., 2023). Thus, lactate metabolism and mitochondrial function in glial cells can affect neuronal discharge frequency. It has been shown that an increase in the number of mitochondria and a disturbance in their dynamics in the hippocampus can lead to excessive excitation of hippocampal neurons and prolonged epileptic duration in mice (Bebensee et al., 2017; Lee et al., 2022) (Figure 1).

Activated microglia can cause neuronal damage by releasing dysfunctional mitochondria, whereas astrocytes can support neuronal survival by transferring functional mitochondria to them (Jia et al., 2023). Astrocytes might regulate neuronal activity by providing “powerhouses” (mitochondria) and the energy substrate lactate, thereby affecting anxiety-like behavior and cognitive deficits caused by seizures (Wu et al., 2023; Jia et al., 2023). Neurons can take up glucose through GLUT3 and break it down via glycolysis to generate energy, but their reliance on astrocytes for energy metabolism may help them avoid apoptosis induced by high glycolytic rates (Wu et al., 2023). Thus, the regulation of neuronal activity by astrocytes is closely related to mitochondrial function and lactate metabolism. Mitochondria have been shown to transfer between neurons and glial cells, and exogenous mitochondria can cross the blood–brain barrier to reduce ROS release induced by epilepsy, thereby reducing hippocampal neuron loss and glial activation (Jia et al., 2023). Numerous studies have confirmed that mitochondrial dysfunction can lead to increased lactate production through enhanced anaerobic glycolysis (Barros et al., 2023; Yang et al., 2024; Wu et al., 2023), and the lactate may mediate the occurrence and development of epilepsy by promoting protein lactylation modifications (Figure 1).

### 2.3 The impact of lactate metabolism on neuronal cell death

Previous studies have suggested that neuronal loss associated with programmed death plays an important role in the development of epilepsy (Liang et al., 2023).

#### 2.3.1 NETosis

Neuroinflammation is one of the main causes of neuron loss (Li et al., 2021). Neuroinflammation is characterized not only by the proliferation of glial cells but also by disruption of the blood–brain barrier and migration of peripheral immune cells into the brain. Neutrophils infiltrate damaged brain tissue immediately after ischemic injury, and they can sustain neuronal damage around the infarct by releasing neutrophil extracellular trapping networks (NETs) (Li et al., 2021; Bernis et al., 2023; Byun et al., 2023). NETosis is a form of suicidal death for neutrophils, characterized by the formation and release of NETs, which primarily consist of double-stranded DNA, histones, and granule proteins (Li et al., 2021). During chronic inflammatory damage in the central nervous system, inhibiting the activity of key enzymes in the glycolytic pathway can block the activation of NETosis by reducing lactate levels (Awasthi et al., 2019; Ye et al., 2022; Wang et al., 2018). NETosis may exacerbate neuroinflammation by releasing inflammatory factors to induce neuronal loss in brain tissues after ischemic infarction. The hypoxic environment created by ischemic stroke can induce the occurrence of glycolysis, leading to the production of large amounts of lactate. The lactate may trigger NETosis and contribute to the development of post-stroke epilepsy by enhancing the lactylation of histones in neutrophils that infiltrate the ischemic focus.

#### 2.3.2 Cuproptosis

**Figure 2:**
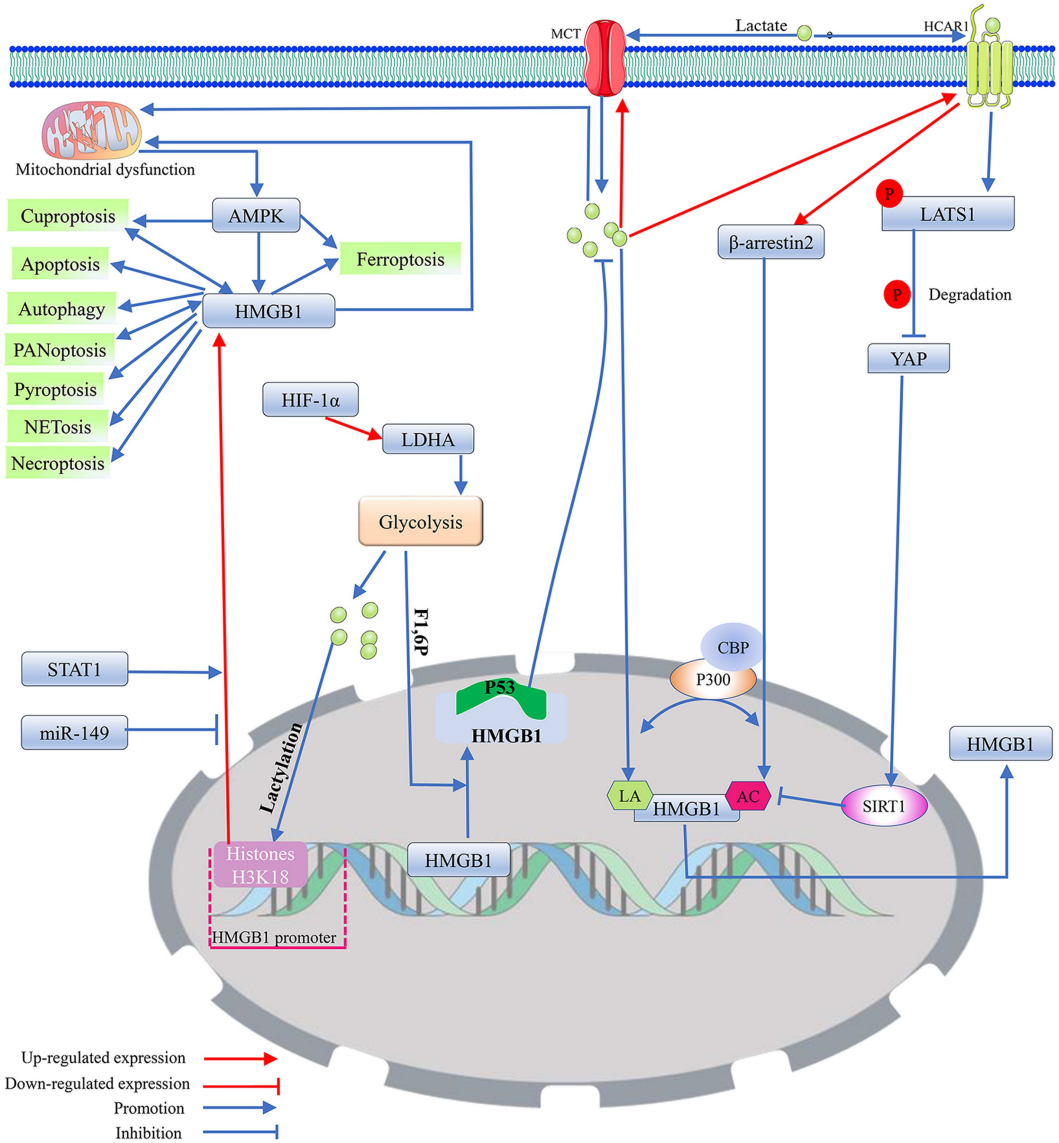
The potential regulatory mechanism of lactate on the biological activity of HMGB1. 1. As a DNA-binding protein, HMGB1 is usually located within the cell nucleus, but when it separates from the DNA strand and migrates out of the cell nucleus, its properties that enhance inflammation and immune responses are activated. Mitochondrial dysfunction promotes the nuclear translocation of HMGB1 through the activation of AMPK, thereby inducing HMGB1 mediated programmed cell death. Among them, cuproptosis and PANoptosis can further promote the nuclear translocation of HMGB1. In addition, miR-149 and STAT1 can affect HMGB1-mediated programmed cell death and mitochondrial dysfunction by regulating the expression level of HMGB1. 2. Exogenous lactate can upregulate β-arresin2 expression by binding to HCAR1, thereby promoting P300/CBP recruitment to the nucleus. P300/CBP promotes HMGB1 secretion and activation by catalyzing HMGB1 lactylation/acetylation. Meanwhile, the binding of exogenous lactate to HCAR1 can upregulate the level of LATS1 phosphorylation, which in turn promotes the phosphorylation and degradation of YAP. The degradation of YAP could promote the acetylation of HMGB1 by reducing SIRT1 activity. In addition, exogenous lactate can be taken up by cells via MCT, thereby mediating mitochondrial dysfunction by promoting protein lactylation, and mitochondrial dysfunction can promote HMGB1 activation. Elevated intracellular lactate levels upregulate the expression of MCT1 and HCAR1. 3. Transcription factor HIF-1α can promote glycolysis by enhancing the expression of LDHA. The lactate produced by glycolysis can not only directly promote HMGB1 lactylation but also enhance the expression of HMGB1 by upregulating the lactylation level of histone H3K18 in its promoter region. F1,6P, as an intermediate product of glycolysis, can bind to the K43/K44 sites of the HMGB1 protein to promote the separation of HMGB1 from DNA in the cell nucleus. The free form of HMGB1 can block P53 degradation by binding to the oncogenic factor P53, thereby reducing the production of lactate. Acetylation (AC); adenosine 5′-monophosphate-activated protein kinase (AMPK); CREB-binding protein (CBP); fructose-1,6-bisphosphate (F1,6P); hypoxia-inducible factor 1 (HIF-1); high-mobility group box 1 (HMGB1); hydroxy-carboxylic acid receptor 1 (HCAR1); lactylation (LA); large tumor suppressor kinase 1 (LATS1); lactate dehydrogenase A (LDHA); monocarboxylate transporter (MCT); signal transducer and activator of transcription 1 (STAT1); silencing regulatory protein 1 (SIRT1); and yes-associated protein (YAP).

Recent research has indicated that neuronal cuproptosis may be an important factor in the initiation and progression of TLE (Yang et al., 2023). Cuproptosis is a mitochondrial proteotoxic stress-dependent mode of regulated cell death (RCD), where the accumulation of copper in cells is crucial (Chen et al., 2023). Ferredoxin 1 (FDX1), a key protein in cuproptosis, can trigger mitochondrial protein lipidization, leading to mitochondrial protein toxicity stress. This form of stress is distinct from copper-induced mitochondrial oxidative stress and the iron-induced oxidative cell death associated with ferroptosis. As mitochondria are the primary targets in cuproptosis, the process includes the disintegration of the mitochondrial membrane and the functional loss of enzymes critical to the tricarboxylic acid cycle (Liu et al., 2022). When oxidative phosphorylation is inhibited, glycolysis is activated as the main energy source for cells (Barros et al., 2023). It seems that the occurrence of cuproptosis may help enhance the level of lactate. In turn, when aerobic glycolysis replaces the aerobic oxidation of sugars as the main energy source for cells, cuproptosis is likely to be suppressed (Xiong et al., 2023). It has been demonstrated that overexpression of FDX1 induces cuproptosis in tumor cells by catalyzing the lipoylation of PDH and α-ketoglutarate dehydrogenase (Schulz et al., 2023). However, HIF-1α can block FDX1-induced cuproptosis by indirectly inhibiting PDH (Chen et al., 2023). PDH promotes the entry of pyruvate into mitochondria and initiates the tricarboxylic acid cycle. Inhibition of PDH activity leads to mitochondrial energy depletion, which activates AMPK to promote cuproptosis (Xue et al., 2023). Copper overload may induce cuproptosis by mediating mitochondrial respiratory chain damage by over-activating the energy sensor AMPK. Cuproptosis can promote the release of the pro-inflammatory mediator High-Mobility Group Box 1 (HMGB1) (Liu et al., 2022; Xue et al., 2023) (Figure 2). It appears that cuproptosis-induced mitochondrial dysfunction may inhibit the exacerbation of cuproptosis by promoting the production of lactate, which has been shown to activate HIF-1α. HIF-1α may antagonize the effects of FDX1-mediated lipid acylation of PDH by inhibiting the activity of PDH to inhibit cuproptosis (Jiang et al., 2021).

#### 2.3.3 Ferroptosis and disulfidptosis

Mitochondria, as the center of cellular energy metabolism, are not only the main target of cuproptosis but also the main site of iron utilization. The accumulation of excessive iron (Fe^2+^) in mitochondria, which is a key factor in triggering ferroptosis, can lead to mitochondrial functional impairment, which exacerbates oxidative stress-induced cellular damage through the release of ROS, destructive lipids, mitochondrial DNA, and proteins (Moos et al., 2023). Ferroptosis is a form of programmed cell death caused by lipid peroxidation associated with energy metabolism (Zhao et al., 2020). The onset and progression of ferroptosis may involve disruption of mitochondrial function, which often induces the Warburg effect (Yang et al., 2023). Elevated lactate concentration in cells may regulate iron ion metabolism by promoting lactylation modification of proteins, thereby promoting cellular ferroptosis (Zhang et al., 2023). Nevertheless, exogenous lactate can assist cells in replenishing their energy resources by activating HCAR1 or MCT1, increasing ATP production. With the enhancement of ATP levels, the potency of AMPK is gradually reduced, resulting in the inhibition of ferroptosis or cuproptosis (Xue et al., 2023) (Figure 2). Mitochondrial dysfunction is not the sole factor inducing the Warburg effect in neurons. In HT22 cells, the activation of small-conductance calcium-activated K^+^ (SK) channels may initiate aerobic glycolysis as the main energy source for the cell and reduce the generation of ROS during the process of mitochondrial energy metabolism to inhibit ROS-induced cellular oxidative stress, thereby preventing the cell from entering ferroptosis (Krabbendam et al., 2020).

With the continuous deepening of research on iron metabolism and ferroptosis in recent years, inhibiting ferroptosis in neuronal cells is considered a potential new strategy for treating epilepsy (Moos et al., 2023; Chen et al., 2020). It has been shown that the neuronal excitotoxicity inducer glutamate can promote ferroptosis by releasing mitochondrial ROS (Krabbendam et al., 2020). Cerebral infarction-induced cerebral ischemia and hypoxia promote the onset of glycolysis and lactate production in neuronal cells by upregulating the expression of LDHA (Yao and Li, 2023). Elevated lactate levels promote lactylation modification of lymphocyte cytoplasmic protein 1 in neuronal cells of Middle cerebral artery occlusion (MCAO) rats, thereby exacerbating cerebral infarction-induced neuronal loss, which is an important cause of post-stroke epilepsy (Zhang et al., 2023).

The neuronal solute carrier family 7 member 11 (SLC7A11)/GPX4 pathway may play a role in reducing the occurrence of epilepsy by inhibiting ferroptosis (Liang et al., 2023; Yuan et al., 2021). SLC7A11, a transport protein that facilitates the uptake of cystine and the efflux of glutamate, is instrumental in activating GPX4. This activation occurs through its role in cystine uptake, essential for the synthesis of glutathione, a key antioxidant that GPX4 uses to prevent lipid peroxidation and, consequently, ferroptosis in neuronal cells (Yang et al., 2023). However, the accumulation of disulfides, such as cystine, in the cell causes disulfide bond stress, which mediates the formation of disulfide bonds between actin cytoskeletal proteins and the breakdown of the actin filament (F-actin) network, ultimately leading to disulfidptosis (Liu et al., 2023). Therefore, the intake of cystine mediated by SLC7A11 can suppress neuronal ferroptosis while also triggering neuronal disulfidptosis. The efficiency of SLC7A11 in transporting cystine is affected by the level of glutamate in the cell. Although HIF-1α promotes the uptake of cysteine mediated by SLC7A11 by upregulating the expression of SLC1A1, lactate may not be involved in the resistance to ferroptosis driven by the glutamate metabolism of SLC7A11 (Yang et al., 2023).

#### 2.3.4 Apoptosis

Recent research has shown that neuronal autophagy and apoptosis related to epilepsy are mediated by the mTOR signaling pathway (Liu et al., 2022), and these two forms of programmed cell death may exist in a complex interplay in the pathological changes leading to neuronal loss (Arab et al., 2023). Caspase-3 is one of the key effector molecules in executing cellular apoptosis. Activated caspase-3 and DNA-binding protein HMGB1 can rapidly transfer to mitochondria and degrade mitochondrial proteins, thereby mediating the loss of hippocampal CA1 and GABAergic interneurons to maintain sustained epilepsy (Kim et al., 2021). It is evident that HMGB1, like caspase-3, can mediate neuronal apoptosis-associated epilepsy by affecting mitochondrial structure and function. Recent studies have suggested that HMGB1 activation is regulated by lactylation modifications and that this process is closely linked to cellular lactate production (Yao and Li, 2023). Lactate promotes mitochondrial hyperfission by mediating an increase in lactylation of the mitochondrial Fission 1 protein (Fis1) lysine 20 (Fis1 K20la), which can contribute to mitochondrial hyperfission by inducing ATP depletion, mitochondrial ROS overproduction, and mitochondrial damage-mediated apoptosis. Conversely, activation of PDH downregulates lactylation of Fis1 K20 by decreasing the level of lactate produced by glycolysis, thereby alleviating the apoptosis-inducing effect of mitochondrial damage (An et al., 2023). Meanwhile, inhibiting H3 histone lactylation at H3K9 and H3K14 sites can induce apoptosis (Xu et al., 2023). It follows that lactate may mediate mitochondrial function-related apoptosis by regulating lactylation modifications of histones and non-histone proteins.

#### 2.3.5 Pyroptosis

Pyroptosis, as an inflammatory mode of programmed cell death, can mediate the expression and release of caspase/Gasdermin D (GSDMD)-associated inflammatory factors through the activation of inflammasomes (Xia et al., 2021). The Nucleotide-binding oligomerization domain-like receptor family card domain-containing protein (NLRP3) inflammasome is an important activator of pyroptosis related to epilepsy, and it has been demonstrated that STAT3 can promote the expression of NLRP3 by catalyzing the acetylation of histone H3K9 on the NLRP3 promoter, thereby activating the NLRP3/caspase-1 pathway and exacerbating the damage to neurons in epileptic mice (Jiang et al., 2021). Epilepsy-related neuronal pyroptosis is initiated by the accumulation of ROS caused by mitochondrial impairment in neurons (Xu et al., 2023). Mitochondrial dysfunction often forces cells to obtain energy through glycolysis. A study on brain ischemia found that lactate upregulates HMGB1 expression by promoting histone lactylation of the HMGB1 promoter, which may lead to neuronal pyroptosis (Yao and Li, 2023). It is closely related to the activation of the NLRP3 inflammasome by HMGB1 (Liu et al., 2022). Thus, lactate may affect the occurrence of epilepsy by regulating neuronal apoptosis.

#### 2.3.6 Autophagy

Autophagy is a process by which cells degrade their own excess or aging organelles and misfolded proteins (Yang et al., 2020). Lactate and pyruvate can activate mitochondrial autophagy and autophagy in primary neurons and astrocytes by lowering intracellular hydrogen ion concentration at non-toxic concentrations, which facilitates the restoration of mitochondrial function for the protection of these cells from apoptosis and necrosis (Fedotova et al., 2021). It has been shown that a lack of neuronal autophagy contributes to epilepsy and that seizures may be further exacerbated by triggering autophagy dysfunction, thus creating a vicious cycle (Ali et al., 2023). Lactate not only protects neurons by regulating mitochondrial autophagy within the cells but also maintains neuronal activity by regulating mitochondrial autophagy in glial cells (Zhu et al., 2022). Moreover, lactate may further promote cytoplasmic acidification by inducing a shift in cellular energy metabolism from oxidative phosphorylation to glycolysis, thereby sustaining mitochondrial autophagy (Komilova et al., 2021). With the exacerbation of neuronal autophagy, the autophagy articulation protein Sequestosome 1 is heavily depleted, which can inhibit the activation of the phosphatidylinositol 3-kinase (PI3K)/protein kinase B (Akt)/mTOR pathway. Decreased mTOR activity impedes the downregulation of the transcription factor Hif-1α, resulting in increased expression of key glycolytic enzymes LDHA and hexokinase-2 (HK2) (Song et al., 2021). In addition, SIRT1, located in the nucleus and cytoplasm, directly or indirectly promotes the formation of autophagic vesicles and improves mitochondrial function, thus blocking epileptogenesis and development (Ali et al., 2023). Because both SIRT1 and lactate can mediate autophagy in neurons to control epileptogenesis, it is likely that there is an association between SIRT1 and lactate metabolism. A recent study has confirmed that lactate can improve long-term cognitive impairment in newborns repeatedly exposed to sevoflurane by activating SIRT1-mediated hippocampal neurogenesis and synaptic remodeling (Qiu et al., 2023).

#### 2.3.7 Necroptosis

Necroptosis, which is a novel pattern of cell death associated with inflammation, is considered one of the complex mechanisms of neuronal death after status epilepticus (Roh et al., 2023). The accumulation of ROS due to mitochondrial damage can promote necroptosis of neurons in the hippocampus of acute epilepsy patients through activation of the RIPK1/RIPK3/MLKL pathway, and mitochondrial enzyme SIRT3 and the indole-derived small molecule NecroX-7 can alleviate seizures by suppressing the production of mitochondrial ROS (Roh et al., 2023; Song et al., 2021). This shows that mitochondria are important organelles that mediate epilepsy-related neuronal necroptosis. The glycolytic product lactate may regulate the activity of HIF-1α to block the release of mitochondrial ROS mediated by PDH, thereby regulating the necroptosis induced by RIPK (Chen et al., 2023; Jiang et al., 2021; Icard et al., 2023; Wong Fok Lung et al., 2020; Weindel et al., 2022; Luo et al., 2022). Furthermore, necroptosis is activated in hippocampal astrocytes and microglia to mediate neuroinflammation 4 h after status epilepticus induction (Wu et al., 2021).

#### 2.3.8 PANoptosis

PANoptosis, which is an inflammatory programmed cell death driven by PANoptosome, has key features of pyroptosis, ferroptosis, apoptosis, and necroptosis (Lin et al., 2022; González-Rodríguez and Fernández-López, 2023). PANoptosis may be present in brain tissue I/R and is a new way of causing neuronal loss (González-Rodríguez and Fernández-López, 2023). Mitochondrial dysfunction likely affects PANoptosis of neurons, as it does most programmed forms of cell death (She et al., 2023). OGD/R induces PANoptosis of neurons by inducing mitochondrial fission and dysfunction (Zeng et al., 2023). It may be related to the hypoxia-induced enhancement of glycolytic metabolism, as several enzymes involved in glycolytic metabolism have been shown to directly regulate mitochondrial functions (Liu et al., 2023). Thus, mitochondrial oxidative stress and leakage of mitochondrial contents induced by multiple factors are important triggers of PANoptosis (Zeng et al., 2023; Liu et al., 2023; Bi et al., 2022). Numerous studies have demonstrated that the structural proteins of the PANoptosome are able to induce epilepsy-associated neuronal loss by mediating crosstalk in the neuronal programmed death pathway (Xia et al., 2023; Shi et al., 2023). PANoptosis may possess characteristics of various programmed cell deaths, including but not limited to pyroptosis, apoptosis, and necroptosis (Lin et al., 2022; González-Rodríguez and Fernández-López, 2023). Although it cannot currently be confirmed that PANoptosis includes cuproptosis, the evidence suggesting that (Fe-S) cluster damage can lead to cuproptosis, ferroptosis, and PANoptosis implies that these three programmed cell death pathways may be closely related to (Fe-S) cluster-mediated mitochondrial dysfunction (Chen et al., 2023; Lin et al., 2022). Therefore, PANoptosis, which possesses characteristics of all these programmed cell death types, may also be regulated by lactate metabolism, such as pyroptosis, apoptosis, and necroptosis.

### 2.4 Mitochondrial dysfunction intervention in the context of epilepsy-related neuronal programmed death

Accumulating evidence indicates that the occurrence and development of epilepsy are closely related to mitochondrial dysfunction (Moos et al., 2023). Damaged mitochondria can mediate many forms of neuronal programmed cell death by releasing mitochondrial contents, such as ROS, mitochondrial DNA, and mitochondria-associated proteins, leading to epilepsy-associated neuronal cell loss (Jia et al., 2023). Mitochondria can play an anticonvulsant and anti-neuroinflammatory role by synthesizing D-fructose 1,6-diphosphate, a glycolysis intermediate that inhibits sugar metabolism (Jia et al., 2023). It has been demonstrated that artificial mitochondrial transplantation may inhibit the loss of hippocampal neurons and the activation of glial cells by ameliorating the metabolic imbalance induced by mitochondrial dysfunction, thereby reducing hippocampal damage after seizures and ameliorating epilepsy-associated psychiatric and cognitive disorders (Jia et al., 2023). Therapeutic interventions aimed at improving mitochondrial function in the central nervous system may effectively alleviate neurological damage and cognitive impairments related to epilepsy. A recent study has demonstrated that honokiol attenuates mitochondrial dysfunction and its associated oxidative stress by regulating mitochondrial ROS homeostasis and that it may stabilize mitochondrial function through activation of mitochondrial DNA transcription mediated by the glutamate receptor N-methyl-D-aspartate receptor/AMPK/peroxisome proliferator-activated receptor γ-coactivator-1α/SIRT3 pathway (Wang et al., 2022). Overexpression of SIRT3 in neuronal cells is protective against ischemia–reperfusion injury in the mouse spinal cord (Gu et al., 2023). Meanwhile, the ketogenic diet-induced overexpression of SIRT1 can maintain the normal physiological function of neurons by promoting neuronal autophagy to eliminate protein aggregates and damaged mitochondria (Dyńka et al., 2022).

Brain injury and energy deficiency promote the transport of energy substrates, such as ketones, lactate, and acetoacetate, into cells by upregulating the number of MCT channels on the neuronal membrane, thereby ensuring normal neuronal function. Astrocytes may reduce glucose availability and frequency of epileptic seizures by producing ketones and shuttling them via MCT channels to neurons (Dyńka et al., 2022). However, some other studies suggest that transporting these energy substances and mitochondria may increase neuronal excitability, thereby triggering epilepsy. The energy substances and mitochondria transferred from glial cells to neurons may lead to neuronal excitation, an important factor in inducing epilepsy (Nagy et al., 2018; Wei et al., 2023; Jia et al., 2023). The increased synthesis of lactate caused by cerebral ischemia promotes mitochondrial apoptotic pathway-mediated neuronal death, which may induce the occurrence of post-stroke epilepsy by upregulating the lactylation levels of key proteins in the calcium signaling pathway, such as Scl25a4 (K245), Slc25a5 (K96), and Vdac1 (K20, K266) (Yao et al., 2023). However, when lactate and ketones replace glucose as the main energy source for neurons, glucose can enter the pentose phosphate pathway and produce a large amount of nicotinamide adenine dinucleotide phosphate, which can offset the oxidative effect of ROS released during the mitochondrial utilization of lactate for production (Mayorga-Weber et al., 2022). During status epilepticus, HMGB1, a non-histone DNA-binding protein, can be transferred from the cell nucleus to the mitochondria, thereby disrupting the energy metabolism balance of the mitochondria (Kim et al., 2021). HMGB1 in microglia can promote the release of inflammatory factors such as IL-1β and IL-18 by activating the NLRP3/NF-κB/MAPKs pathway, leading to the activation of the NLRP3 inflammasome in hippocampal neurons, which can subsequently lead to pyroptosis and apoptosis (Liu et al., 2022; Shen et al., 2018). The neuronal injury promotes the release of neurally mediated ATP, which reactivates the NLRP3/NF-κB/MAPKs pathway via binding to the purinergic receptor P2X ligand-gated ion channel 7 in microglia without HMGB1. HMGB1 may be a central element in the tandem of neuronal death and glial cell activation in the hippocampus, and HMGB1 can amplify the effects of neuroinflammatory injury by promoting the interaction between neuronal death and glial cell activation (Liu et al., 2022; Shen et al., 2018) (Figure 1).

### 2.5 Regulation of neuronal programmed death by HMGB1

#### 2.5.1 HMGB1 and mitochondria

HMGB1 nucleus-to-cytoplasm translocation has been shown to maintain status epilepticus and drug-resistant temporal lobe epilepsy by mediating oxidative stress in brain tissue in neurons and glial cells (Pauletti et al., 2019). The function and activity of HMGB1 are determined not only by its subcellular localization but also by its oxidation–reduction status and post-translational modifications (Chen et al., 2023). Mitochondrial damage-mediated oxidative modification of HMGB1 promotes cytoplasmic accumulation and extracellular secretion of HMGB1. HMGB1 secreted into the extracellular compartment initiates the expression of NF-κB-mediated inflammatory factors (e.g., TNF-α and IL-1β) through activation of Toll-like receptor 4 (TLR4) on the cell surface, ultimately leading to inflammatory tissue damage (Zhao et al., 2023). Brain tissue ischemia induces HMGB1 translocation from the cell nucleus to the mitochondria and extracellular region in neurons and astrocytes, leading to neuronal apoptosis associated with epileptic status (Wang et al., 2020; Dai et al., 2023). During this process, the transposition of HMGB1 triggers the destruction of mitochondria, and the released contents of mitochondrial lysis further intensify HMGB1-mediated neuroinflammatory damage (Wang et al., 2020; Dai et al., 2023; Zhang et al., 2020) (Figure 2).

#### 2.5.2 HMGB1 and various types of programmed cell death

**Figure 3:**
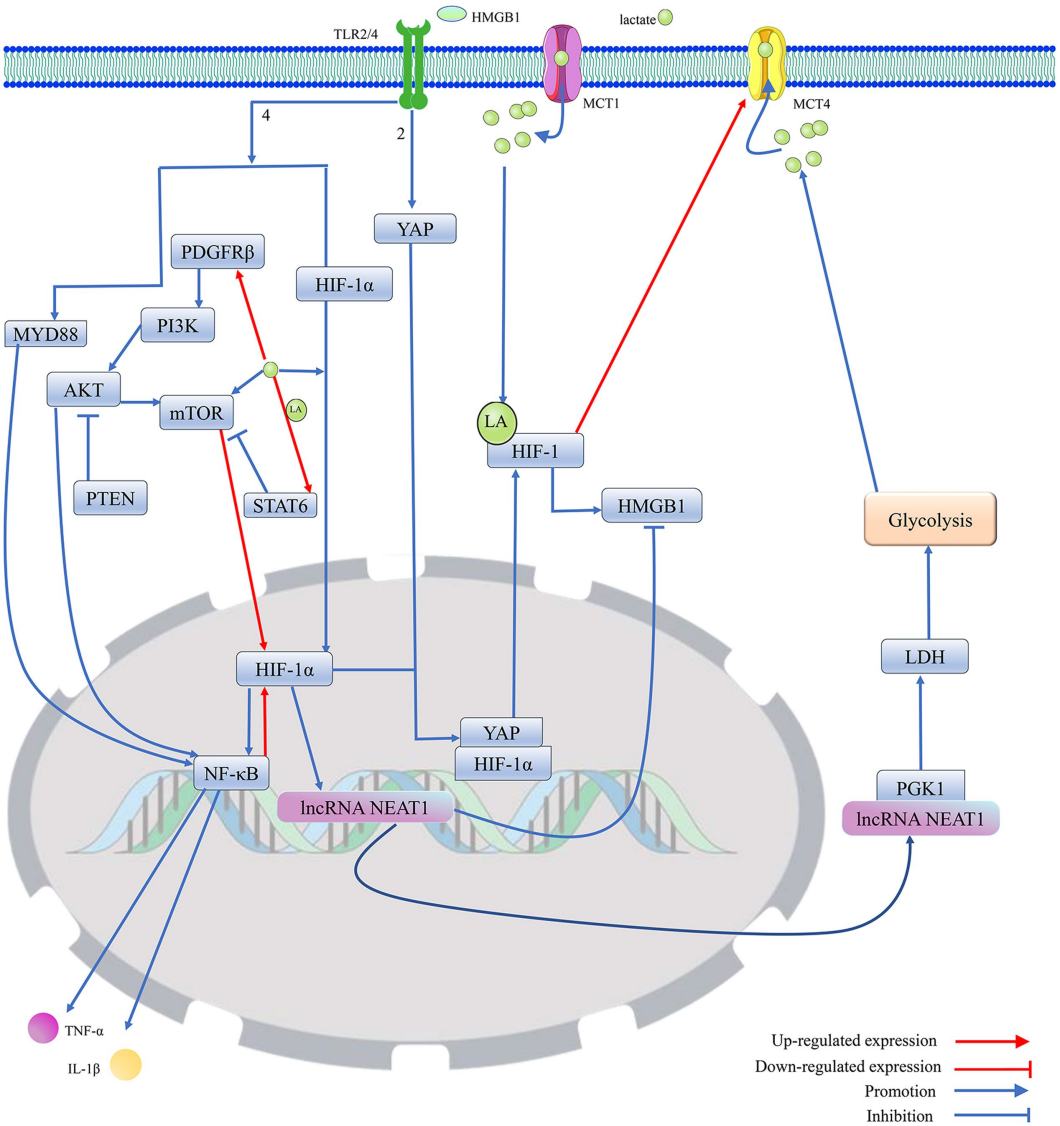
Protein lactonylation modification is an important influence in the process that is HIF-1-mediated HMGB1 activity. 1. Lactate, as a metabolite of cellular adaptation to hypoxic environments, is mainly produced by glycolysis. MCT1 and MCT4 are mainly responsible for the cellular uptake and release of lactate. Elevated intracellular lactate levels not only promote the translocation of HIF-1α from the cytoplasm to the nucleus to regulate gene expression but also stabilize the structure and enhance the biological activity of HIF-1 by promoting HIF-1 lactylation. Furthermore, lactate can promote the expression of HIF-1α by directly or indirectly enhancing the activation of mTOR. However, lactate can upregulate STAT6 expression by promoting histone lactylation, thereby blocking PDGFRβ/PI3K/AKT/mTOR-mediated HIF-1 expression. 2. Activated HIF-1 promotes the secretion and activation of HMGB1, which is secreted into the extracellular compartment and promotes the migration of YAP from the cytoplasm to the nucleus by binding to TLR2, thereby promoting the activation of HIF-1. Meanwhile, HMGB1, by binding to TLR4, not only directly promotes the migration of HIF-1 to the nucleus to exert transcriptional regulation but also activates the MYD88/NF-κB pathway to enhance the expression of HIF-1. However, HIF-1α can reduce the expression of HMGB1 by upregulating the transcript level of lncRNA NEAT1, thereby blocking the excessive activation of HIF-1 induced by HMGB1. The lncRNA NEAT1 can stabilize the structure of PGK1 by binding to PGK1, which mediates the glycolysis/lactate/HIF-1α pathway by increasing LDH activity, but HIF-1 can promote lactate efflux by upregulating the expression of MCT4, which blocks the excessive activation of HIF-1 induced by lactate. Furthermore, PTEN could block HIF-1 expression, which is mediated by the PDGFRβ/PI3K/AKT/NF-κB pathway, by inhibiting AKT, thereby reducing HMGB1-induced release of cellular inflammatory factors. Protein kinase B (AKT); hypoxia-inducible factor 1 (HIF-1); high-mobility group box. 1 (HMGB1); interleukin 1β (IL-1β); lactylation (LA); lactate dehydrogenase (LDH); monocarboxylate transporter (MCT); mechanistic target of rapamycin (mTOR); myeloid differentiation factor 88 (MYD88); nuclear enriched transcript 1 (NEAT1); platelet-derived growth factor receptor β (PDGFRβ); phosphoglycerate kinase 1 (PGK1); phosphatidylinositol 3-kinase (PI3K); phosphatase and tensin homolog (PTEN); signal transducer and activator of transcription 6 (STAT6); toll-like receptor (TLR); tumor necrosis factor-α (TNF-α); and yes-associated protein (YAP).

Mitochondrial dysfunction can induce epileptogenesis by mediating multiple programmed cell deaths in neurons, and HMGB1 may intervene in epilepsy-associated multiple programmed cell deaths by modulating mitochondrial function. It has been demonstrated that HMGB1 released into the extracellular space can act as a damage-associated molecular pattern protein to mediate a variety of cellular programmed deaths (Chen et al., 2023; Tang et al., 2023). HMGB1, which is secreted extracellularly, may drive the disease process in epilepsy by inducing neuroinflammation and neuronal loss during the silent phase following the initial triggering event (Rosciszewski et al., 2019). HMGB1 transferred from the nucleus to the cytoplasm may activate the Myeloid differentiation factor 88 (MyD88)/NF-κB axis by binding to TLR4. The MyD88/NF-κB axis can induce neurological deficits by promoting neuronal apoptosis, autophagy, and the release of inflammatory factors, such as IL-1β and TNF-α (Lei et al., 2022; Fu et al., 2023; Shang et al., 2020) (Figure 3). STAT1 may upregulate the expression level of HMGB1 by binding to the HMGB1 promoter, thereby affecting the biological effects mediated by the TLR4/MyD88/NF-κB pathway (Fu et al., 2023). In the MCAO model, impaired neurons induce NETosis by releasing large amounts of HMGB1, which induces NETosis and can even be extruded as part of the NET. The extruded HMGB1 may further induce NETosis and platelet activation, which is one of the important reasons for the elevation of HMGB1, thereby exacerbating neuronal inflammatory damage (Kim and Lee, 2020). miR-149 may reduce the occurrence of epilepsy by inhibiting HMGB1-mediated neuroinflammatory injury (Kwak et al., 2023; Parsons et al., 2022; Wang et al., 2022). Cuproptosis and PANoptosis may further induce the release of inflammatory factors and programmed cell death by promoting the release of HMGB1 (Liu et al., 2022; Kwak et al., 2023). In addition, ATP depletion and calcium ions can upregulate the level of HMGB1 phosphorylation through activation of AMPK, which can promote the translocation and activation of HMGB1 (Liu et al., 2022). It cannot be excluded that HMGB1 may induce various forms of programmed death in neurons by regulating mitochondrial function (Figure 2).

Neuroglia may be involved in HMGB1-mediated neuronal programmed cell death (Rosciszewski et al., 2019). HMGB1 induces NF-κB pathway activation in glial cells by activating signaling pathways involving TLR2, TLR4, and the receptor for advanced glycation end products (RAGE). Since activation of the NLRP3/NF-κB/MAPKs pathway in microglia is an important mechanism for HMGB1-induced hippocampal neuronal pyroptosis and apoptosis (Liu et al., 2022; Shen et al., 2018), the limited activation of NF-κB in astrocytes can lead to partial suppression of neuron loss in the absence of microglia, thereby producing a beneficial effect on controlling the occurrence of epileptic seizures (Cai and Lin, 2022; Rosciszewski et al., 2019). The damaged neurons can further promote the activation of microglia by secreting HMGB1 (Kitaoka et al., 2023). HMGB1 secreted by neurons can also directly act on astrocytes, thereby activating NF-κB p65-mediated inflammatory cascade effects (Kaya et al., 2023). It can be speculated that the intervention of glial cells may exacerbate the neuronal death effect induced by HMGB1.

#### 2.5.3 The impact of lactate metabolism on the function of HMGB1

Research has shown that the increased protein lactylation level of hippocampal neurons is accompanied by upregulation of the HMGB1 expression level (Xie et al., 2023). Extracellular lactate can regulate cell death and immune cell polarization by binding to HCAR1 and transport protein MCT on the membrane (Zhai et al., 2022). Lactate primarily relies on the acetylase P300/CBP to introduce the lactide moiety into the lysine residue of HMGB1, thus directly participating in the lactylation of HMGB1. Meanwhile, exogenous lactate specifically increases the expression of β-arrestin2 by binding to HCAR1, which can catalyze the lactylation/acetylation of HMGB1 by facilitating the recruitment of P300/CBP to the nucleus, thereby mediating the secretion of HMGB1. Exogenous lactate promotes the phosphorylation and degradation of Yes-Associated Protein (YAP) by binding to HCAR. YAP can preserve SIRT1 expression and activation, and lactate-mediated degradation of YAP promotes HMGB1 acetylation by reducing SIRT1 activity. Lactate can induce the lactylation of HMGB1 in macrophages and promote its release from these cells (Yang et al., 2022). Fructose-1,6-bisphosphate, an intermediate product of glycolysis, serves as a novel HMGB1 ligand that promotes the segregation of HMGB1 from DNA in the nucleus by directly binding to K43/K44 on the HMGB1 protein. The free HMGB1 prefers to bind to P53 to form the HMGB1-P53 complex, which effectively prevents the degradation of P53, thus blocking glycolysis and removing damaged mitochondria (He et al., 2023; Li et al., 2023). Based on numerous reports that HMGB1 is an important gene in the regulation of glucose metabolism and mitochondria can regulate the activation of HMGB1, it is suggested that HMGB1 may be an important signaling factor related to mitochondrial dysfunction associated with protein lactylation modification-induced cell death (Li et al., 2023; Du et al., 2022; Liu et al., 2022) (Figure 2).

## 3 Regulating the level of protein lactylation may be a measure to intervene in the occurrence and development of epilepsy

Numerous studies have confirmed that histone acetylation is involved in regulating various cellular physiological activities (Table 1). Mitochondrial dysfunction often promotes the preferential catabolism of glucose by aerobic glycolysis to cause the accumulation of lactate, which can regulate cellular gene expression in the form of epigenetic modifications through regulating histone lactylation (He et al., 2023). Indeed, there exists a mutually promoting relationship between protein lactylation and mitochondrial dysfunction; mitochondrial dysfunction is a core element in the pathogenesis of epilepsy (Moos et al., 2023), and the disease process may be affected by protein lactylation.

**Table 1:** The common sites of histone lactylation.

Associated diseases	Subjects	Sites	Lactylation-mediated biological effects	References
Prostate cancer	Prostate cancer cells	H3K18	It may trigger an adenocarcinoma-to-neuroendocrine transition.	He et al. (2023)
Ocular melanoma	Ocular melanoma cell lines (OCM1 and OM431)	H3K18	It can reduce the expression levels of Period Circadian Protein 1 and P53 in ocular melanoma cells by promoting the transcription of YTH Domain Family Protein 2, which can promote cell proliferation and migration.	Yu et al. (2021)
Colon cancer	Tumor-infiltrating myeloid cells	H3K18	It can activate the Janus Kinase 1-STAT3 signaling pathway by upregulating the expression of methyltransferase-like 3 (METTL3) in Tumor-infiltrating myeloid cells for promoting tumor growth.	Xiong et al. (2022)
Ulcerative Colitis	Bone marrow-derived macrophages (BMDMs)	H3K18	It can suppress M1 macrophage polarization and macrophage pyroptosis.	Sun et al. (2021)
Developmental abnormalities of the nervous system	Neural stem/progenitor cells (NSPCs)isolated from E13.5 mice forebrain 2. P19 embryonic carcinoma (EC) cells	H3K18	It can encourage P19 EC cell-derived NSPC to exit the cell cycle and differentiate into neurons.	Dai et al. (2022)
Malignant pleural effusion (MPE)	Human Natural killer T-like cells	H3K18	Upregulation of the H3K18la level in the promoter region of the FOXP3 gene can reduce the anti-tumor function of Natural killer T-like cells by promoting FOXP3 expression.	Wang et al. (2023)
Intestinal inflammation	Th17 cells	H3K18	It may promote Foxp3 expression through upregulation of ROS-driven IL-2 secretion, which stimulates the pro-inflammatory effects of macrophages.	Lopez Krol et al. (2022)
Sepsis	THP1 cells	H3K18	It can drive M2 polarization of Macrophages by increasing the expression of M2 markers (such as Arg1).	Ma et al. (2022)
pulmonary hypertension	Pulmonary artery smooth muscle cells	H3K18	It can promote cell proliferation and vascular remodeling.	Chen et al. (2023)
Uveal melanoma (UM)	Human uveal melanoma cells (92.1)	H3K18	It may inhibit tumor cell growth by promoting the expression of oxidative phosphorylation-related genes and cell growth quiescent markers.	Longhitano et al. (2022)
Clear cell renal cell carcinoma (ccRCC)	Immortalized renal epithelial cells, Von Hippel–Lindau inactive RCC cell lines, and RCC cell lines with endogenous Von Hippel–Lindau	H3K18	It can promote the proliferation and migration ability of ccRCC cells.	Yang et al. (2022)
Cerebral ischemia	N2a cells	H3K18	It can promote N2a cell scorching by upregulating the HMGB1 level.	Yao and Li (2023)
Alzheimer’s disease	1. BV2 cells 2. Hippocampus tissue of AD model mice	H3K18	It can mediate neuroinflammation through direct stimulation of the NF-κB pathway to cause neurodegenerative aging-related disorder characterized by progressive cognitive impairment.	Wei et al. (2023)
Liver cancer	Liver cancer stem cells	H3K9 and H3K56	It can promote tumorigenesis.	Pan et al. (2022)
	The human HCC cells	H3K9 and H3K14	It can block apoptosis in tumor cells.	Xu et al. (2023)
Non-Small Cell Lung Cancer	NSCLC cell lines A549 and H1299	H4K8	It can inhibit the proliferation and migration of NSCLC cells by upregulating the expression of IDH3G and downregulating the expression of HK-1.	Jiang et al. (2021)
Alzheimer’s disease (AD)	Microglia from 5XFAD mice	H4K12	It can stimulate microglial pro-inflammatory activation by promoting the expression of glycolytic genes.	Pan et al. (2022)

### 3.1 Mechanisms involved in influencing the level of protein lactylation

**Figure 4:**
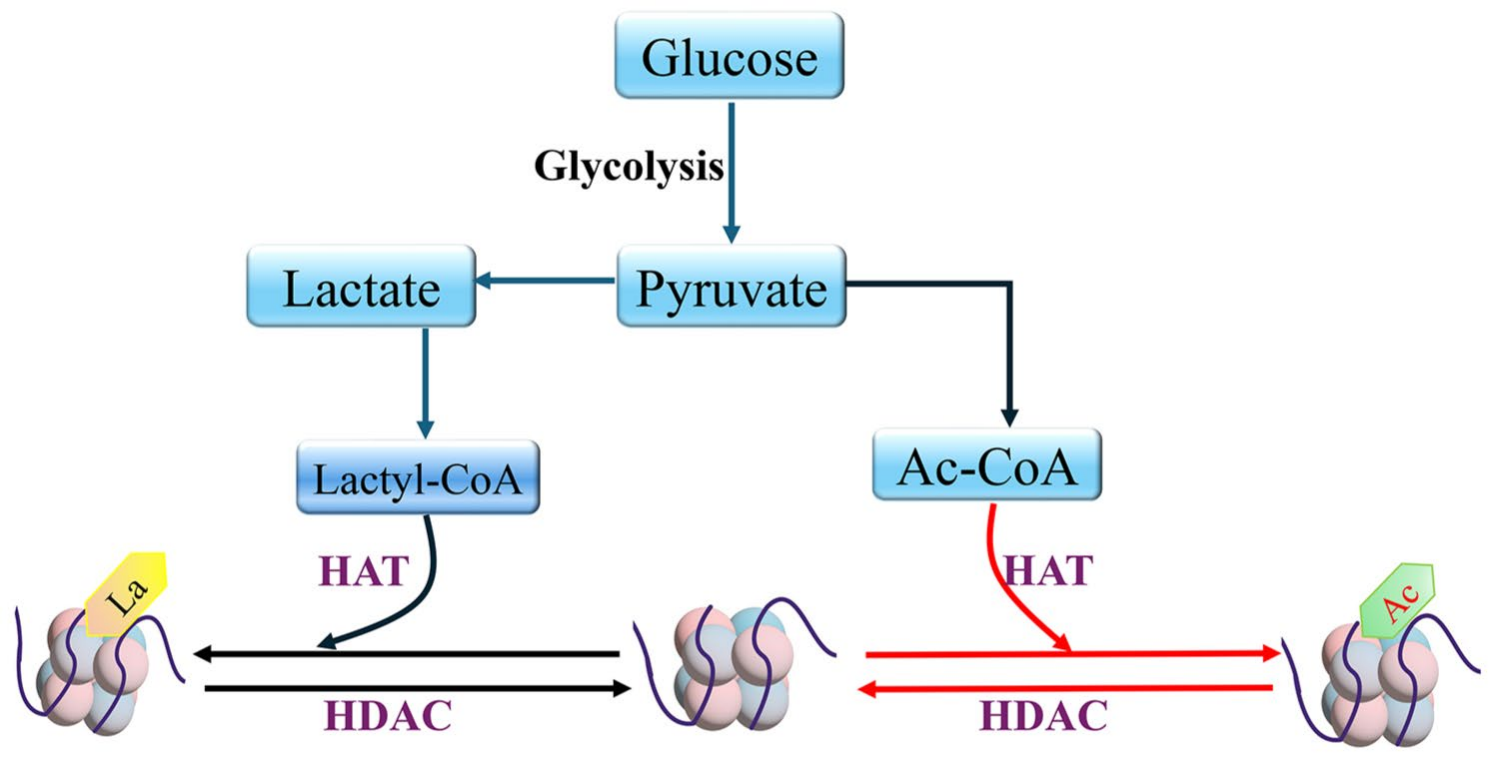
HDAC and HAT mediate histone lactylation and histone acetylation Lactyl coenzyme A (Lactyl-CoA); Acetyl-CoA (Ac-CoA).

Since lactate can be catalyzed to produce lactyl coenzyme A, which has been confirmed as a direct precursor of the lactyl group, factors that affect lactate synthesis and transport may be involved in the regulation of protein lactylation modification (Yang et al., 2022; Dong et al., 2022) (Figure 4).

#### 3.1.1 The impact of proteins related to glucose metabolism on lactate production

Lactate is mainly derived from cellular glycolytic metabolism, and GLUT transfers glucose from the extracellular environment into the cell, thus providing the raw material for lactate production (Fan et al., 2023). HK2 catalyzes the conversion of glucose to glucose-6-phosphate, thereby driving the process of generating lactate (Rho et al., 2023). However, HK1 has been shown to be downregulated by lactate expression, thereby inhibiting the glycolytic pathway (Jiang et al., 2021). Phosphofructokinase-1 and phosphoglycerate kinase 1 (PGK1) progressively convert glucose-6-phosphoglycerate to 3-phosphoglycerate. Fructose-2,6-bisphosphatase 3 is a constitutive activator of phosphofructokinase-1, a key enzyme in glycolysis, and downregulation of fructose-2,6-bisphosphatase 3 and phosphofructokinase-1 expression in astrocytes reduces the production of lactate (Lv et al., 2015).

**Figure 5:**
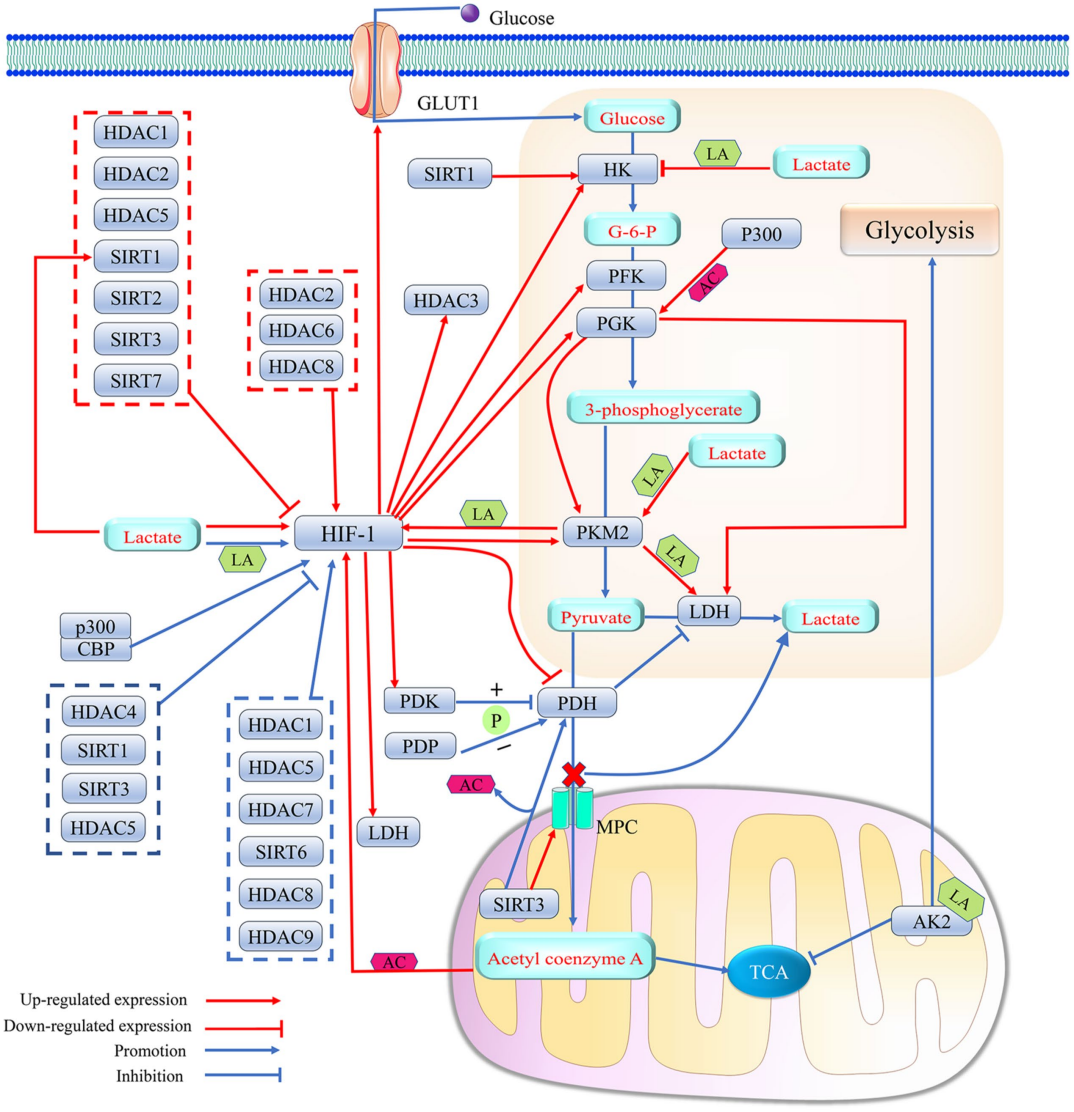
Histone deacetylase affects lactate metabolism by regulating HIF-1-mediated metabolic reprogramming effects. 1. HIF-1, as a transcription factor, enhances lactate production by regulating the expression levels of several key enzymes in glycolysis (HK, PGK, PFK, LDH, PDH and PKM2) and the glucose transport protein GLUT1. PDH promotes the entry of pyruvate into mitochondria via MPC and its conversion to acetyl coenzyme A, which initiates TCA by inhibiting the activity of LDH. HIF-1 catalyzes the phosphorylation of PDH by promoting the expression of PDK, thereby attenuating the activity of PDH. Elevated lactate levels in cells can promote lactylation modification of HIF-1α, which can elevate the transcription factor activity of HIF-1 to further enhance the efficiency of the glycolytic response. Histone acetylation or lactylation of the promoter of HIF-1 can promote HIF-1 expression. The level of promoter histone acetylation of HIF-1 is affected by the concentration of acetyl coenzyme A in cells, and the degree of HIF-1 lactylation can be regulated with PKM2. Meanwhile, the expression levels of both PKM2 and LDH can be upregulated by their promoter histone lactylation. A positive feedback loop consisting of lactate/PKM2/HIF-1/LDH further promotes protein lactylation. In addition, lactylation of the mitochondrial protein AK2 further promotes glycolysis and inhibits TCA. However, the positive feedback regulation of HIF-1α by lactate is not uncontrolled, and high lactate concentrations may block HK expression by upregulating histone lactylation levels in the HK promoter, thereby slowing down the onset of cellular glycolysis. Meanwhile, both PDP-mediated PDH dephosphorylation and SIRT3-catalyzed PDH deacetylation were able to produce less lactate by increasing PDH activity. 2. Histone acetylase P300 upregulates the expression level of PGK1, a key enzyme in glycolysis, by catalyzing its promoter histone acetylation. PGK1 promotes glycolysis by upregulating the expression of LDH and PKM2. Histone acetylase P300/CBP elevates the transcriptional activity of HIF-1 by promoting HIF-1 acetylation. 3. HDACs can intervene in lactate metabolism by regulating HIF-1 activity and/or expression levels. SIRT1 can even promote lactate production by directly upregulating the expression level of HK, while SIRT3 can help more pyruvate enter the mitochondria rather than converting it into lactate by promoting the expression of MPC. HIF-1 can act as a transcription factor to promote HDAC3 expression. Acetylation (AC); adenylate kinase 2 (AK2); glucose-6-phosphate (G-6-P); glucose transporter 1 (GLUT1); hexokinase (HK); histone acetylase P300; histone deacetylase (HDAC); hypoxia-inducible factor 1 (HIF-1); lactic dehydrogenase (LDH); lactylation (LA); mitochondrial pyruvate carrier (MPC); phosphorylation (P); pyruvate dehydrogenase kinase (PDK); pyruvate dehydrogenase (PDH); pyruvate dehydrogenase phosphatase (PDP); phosphoglycerate kinase (PGK); pyruvate kinase isozymes M2 (PKM2); phosphofructokinase-1 (PFK); silencing regulatory protein (SIRT); and tricarboxylic acid cycle (TCA).

Knockdown of PGK1 inhibits the M1 polarization of microglia, thereby preventing the alleviation of neuroinflammation by disrupting glycolytic metabolism in the MCAO rat model (Cao et al., 2023). The enzyme 3-phosphoglycerate is transformed into pyruvate by pyruvate kinase, which has two structural isoforms: PKM1 and PKM2. PKM1 consistently functions as a high-activity tetramer, while PKM2 can exist either as a low-activity dimer or a high-activity tetramer. The low-activity dimer form of PKM2 promotes the conversion of pyruvate to lactate, playing a significant role in driving glycolysis. In contrast, the high-activity forms of both PKM2 and PKM1 facilitate the tricarboxylic acid cycle. A recent study has shown that an increase in the expression of PKM2 and a higher PKM2/PKM1 ratio promote glycolysis (Zhang et al., 2017). In the nervous system, the knockout of PKM2 can result in the compensatory overexpression of PKM1, which may promote the transformation of the energy metabolism of microglial cells from glycolysis to oxidative phosphorylation (Pan et al., 2022). PDH and LDH are key enzymes in determining the fate of pyruvate, an intermediate product of glycolysis, and PDH promotes the conversion of pyruvate to acetyl-coenzyme A, while LDH can shift the metabolism of pyruvate toward the production of lactate (Yang et al., 2022; Jia et al., 2021; Icard et al., 2023). LDH contains two subtypes, LDHA and LDHB. LDHA prefers to convert pyruvate into lactate, while LDHB tends to convert lactate back into pyruvate. However, downregulation of both LDHA and LDHB inhibits glycolysis and reduces lactate production (Yang et al., 2022). Pyruvate Dehydrogenase Phosphatase 1 (PDP1) and PDK control the rate of pyruvate transformation into acetyl-CoA by regulating the activity of PDH. PDK inhibits PDH activity by promoting the phosphorylation of serine 293 on PDH, while PDP1 normally catalyzes PDH dephosphorylation to activate PDH (Chen et al., 2023; Karagiota et al., 2023; Kantarci et al., 2020). Pyruvate transport to the mitochondria is normally blocked by the upregulation of PDK, leading to the production of lactate (Kantarci et al., 2020). In addition, lncRNA Nuclear Enriched Transcript 1 (NEAT1) can increase the activity of PGK1 and inhibit the degradation of PGK1, thereby promoting glycolysis (Liang et al., 2022) (Figures 3, 5).

Previous studies have confirmed possible interactions between key enzymes of glycolytic metabolism. PGK1 accelerates lactate synthesis in microglia by upregulating the expression of PKM2 and LDHA (Cao et al., 2023). The dimer PKM2 can enter the nucleus to form a complex with prolyl hydroxylase 3, thereby promoting the expression of HIF-1α and its target genes (LDH, GLUT1, and PDK1) (Awasthi et al., 2019). Simultaneously, PKM2 may promote the generation of lactate by promoting glycolysis, which can increase the transcript levels of HIF-1α, PKM2, and LDHA through upregulation of H4K12la levels on the HIF-1α, PKM2, and LDHA promoter, thus promoting the further production of lactic acid (Pan et al., 2022). In addition, the knockdown of mitochondrial pyruvate carrier protein 1 promotes the conversion of pyruvate to lactate by blocking the entry of pyruvate into mitochondria (Gao et al., 2023). In turn, lactate reduces the transcription of HK1 by upregulating histone lactylation in the HK-1 promoter, which can inhibit glycolysis (Jiang et al., 2021).

Furthermore, lactate can indirectly regulate its own production by affecting the activity and transcriptional levels of key enzymes in the tricarboxylic acid cycle, such as ATP citrate lyase 2 and IDH3G (Jiang et al., 2021; Yang et al., 2023). Adenylate kinase 2, an ATP-metabolizing enzyme located in the mitochondria, also plays a role in this regulatory network. A specific variant, adenylate kinase 2 K28Ia, promotes glycolysis (Yang et al., 2023) (Figure 5).

#### 3.1.2 Effect of HIF-1 on lactate metabolism

HIF-1, which contains both HIF-1α and HIF-1β subunits, is often activated under hypoxic conditions. HIF-1α, which is the main subunit responsible for functional regulation, often helps cells adapt to hypoxia by regulating the expression of some genes (Liang et al., 2023). HIF-1 affects lactate supply by regulating the expression of glycolysis-related proteins, such as GLUT1, HK2, PFK, PGK1, PKM, LDHA, and PDK (Chen et al., 2023; Jiang et al., 2021; Chen et al., 2023; Eschricht et al., 2015; Hu et al., 2019). There may be positive feedback between lactate and HIF-1 (Jiang et al., 2021; Hu et al., 2019). HIF-1 not only enhances cellular lactate production by promoting the expression of LDHA but also promotes lactate excretion through the upregulation of MCT4, which can maintain the continuity of glycolysis by avoiding the competitive inhibition of LDHA mediated by lactate excess (Huang et al., 2022). Additionally, HIF-1α induces the shift of energy metabolism from oxidative phosphorylation to glycolysis by downregulating the expression of PDH (Lu et al., 2022). HIF-1 can upregulate the expression of PDK and LDHA (Karagiota et al., 2023). PDK prevents pyruvate from being converted to acetyl-coenzyme A by catalyzing the phosphorylation of PDH, which may allow LDHA to convert pyruvate to lactate (Zhang et al., 2019; Chen et al., 2023; Huang et al., 2022). Knockdown of HCAR1 can block lactate-mediated cellular physiological activities by downregulating the transcriptional level of MCT (Wagner et al., 2015). HIF-1 and lactate have been shown to regulate lactate-mediated cellular physiological activities by upregulating MCT and HCAR1 expression (Longhitano et al., 2022; Huang et al., 2022). It is thus clear that HIF-1 can intervene in protein-lactylation-mediated biological effects by regulating the expression of functional proteins related to lactate metabolism and transport (Figure 5).

### 3.2 The impact of HIF-1 on epilepsy

Ischemia/hypoxia-induced elevation of HIF-1α expression in brain tissue can mediate cognitive deficits associated with hippocampal neuronal loss by inducing mitochondrial structural damage and upregulating pyroptosis-associated effectors, TNF-α and IL-1β (Zhao et al., 2022). HIF-1α/meme oxygenase 1 may promote the development of epilepsy by inducing ferroptosis of hippocampal neurons and reducing the activity of antioxidant enzymes in hippocampal tissue (Liang et al., 2023). Numerous studies have suggested that cellular ferroptosis may be mediated by mitochondrial dysfunction and HMGB1 (Yang et al., 2023; Chen et al., 2023; Tang et al., 2023; Ge et al., 2023; Yuan et al., 2022). It can be inferred that HIF-1α may be involved in HMGB1-mediated mitochondrial dysfunction and programmed cell death associated with epilepsy.

#### 3.2.1 HIF-1α and HMGB1

HIF-1α inhibits the expression of HMGB1 by upregulating the transcription level of lncRNA NEAT1, thereby reducing cell apoptosis and inflammatory damage (Luo et al., 2022). Indeed, HIF-1α, which may act as an upstream regulator of HMGB1, can affect the biological effects of HMGB1 by regulating its transcription level. Meanwhile, HIF-1α promotes the production of lactate from glycolysis by upregulating the expression level of LDHA, which exacerbates HMGB1-mediated ischemic neuronal pyroptosis by upregulating the histone H3K18la in the HMGB1 promoter (Yao and Li, 2023). Cell death usually favors the release of HMGB1 into the extracellular compartment. Extracellular HMGB1 can promote the expression of NF-κB and HIF-1α via binding to the TLR4 receptor (Peng et al., 2021). Lactate can enhance the translation level of NF-κB by promoting the translocation of the transcription factor HIF-1α into the nucleus (Peng et al., 2021). NF-κB located in the nucleus not only promotes the expression of inflammatory cytokines (IL-1β and TNF-α) but also enhances HMGB1 activity by elevating HIF-1α expression (Peng et al., 2021; Song et al., 2020). Thus, HIF-1α can regulate the function of HMGB1 through positive feedback. HMGB1 acts as a ligand for the cell surface receptor TLR2 to trigger YAP and HIF-1α nuclear translocation and activate the Hippo-YAP/HIF-1α pathway. Activated YAP not only promotes the expression of HIF-1α but also maintains HIF-1α stability by binding to HIF-1α in the nucleus, thereby promoting the expression of target genes of HIF-1α. Conversely, knockdown of HIF-1α may inhibit HMGB1 by reducing YAP nuclear translocation. It further confirms that HIF-1α can affect cell death mediated by HMGB1 (Zhang et al., 2019) (Figure 3).

#### 3.2.2 HIF-1α and mitochondria

**Figure 6:**
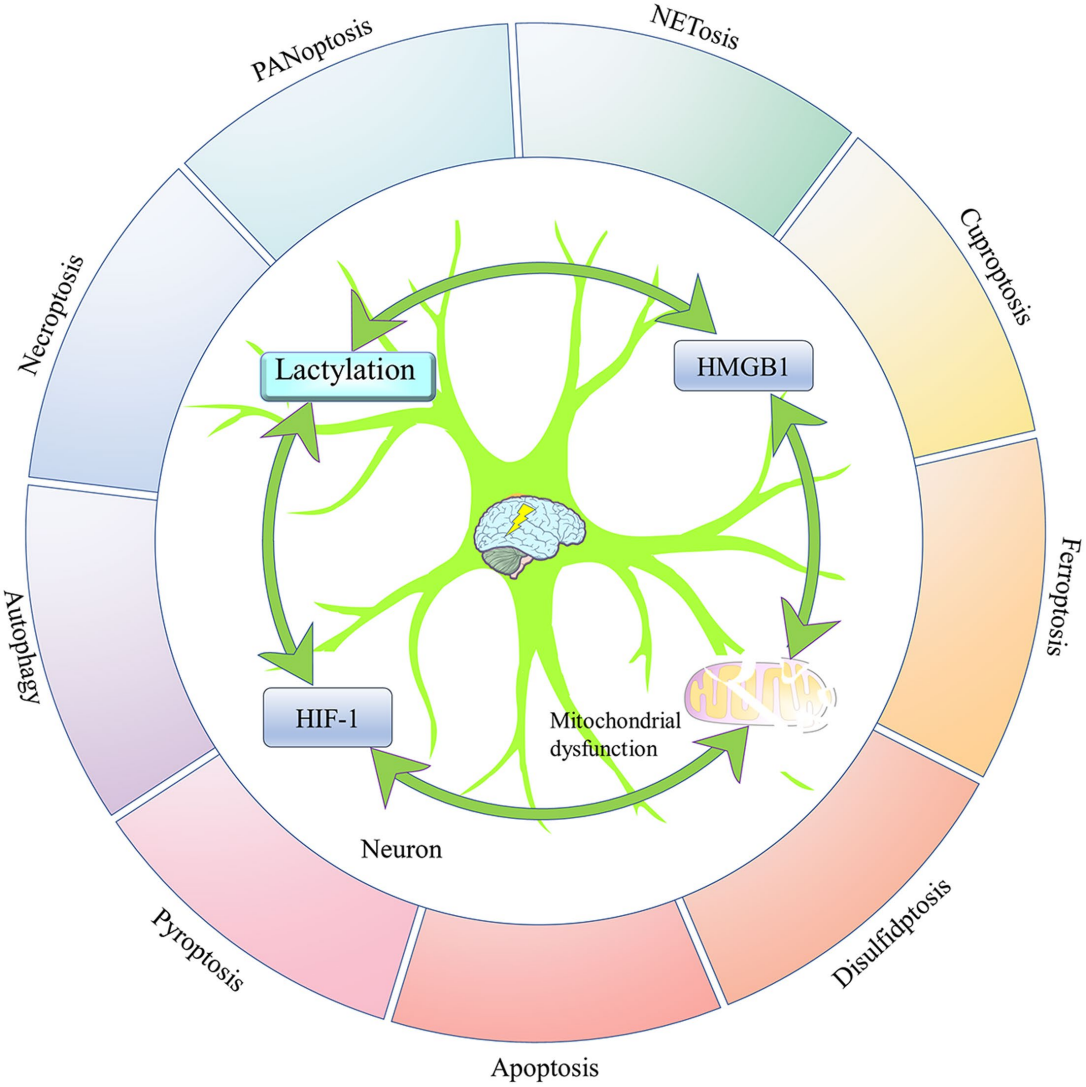
The four elements mediating epilepsy-related neuronal death. Neuronal death is one of the important pathological changes in epileptogenesis and development. Lactylation modification, HIF-1, and mitochondrial dysfunction all intervene in epilepsy-associated neuronal death by regulating the activity of HMGB1, which is a central factor in the regulation of multiple programmed cell deaths. In addition, there is an interactive relationship between lactylation modification, HIF-1, and mitochondrial dysfunction. Hypoxia-inducible factor 1 (HIF-1); and high-mobility group box 1 (HMGB1).

HIF-1α shifts energy production from oxidative phosphorylation to glycolysis by promoting the transcription of related genes, which reduces ROS production to reverse the mitochondrial dysfunction induced by oxidative stress (Li et al., 2019). Although HIF-1α does not stabilize mitochondrial function by promoting the transcription of mitochondrial DNA as SIRT3 does (Wang et al., 2022), HIF-1α, as a protein regulating iron homeostasis, decreases the partial pressure of oxygen, translocates to the mitochondria, and acts directly on the mitochondrial respiratory chain complex in hypoxia, thereby blocking the sustained effects of mitochondrial ROS on mitochondrial function and ultimately achieving multi-modal neuroprotection (Fouché et al., 2023). In addition, HIF-1α may exert cytoprotective effects by mediating mitochondrial autophagy (Lestón Pinilla et al., 2021; Fu et al., 2020). However, intermittent hypoxia induces cellular mitochondrial dysfunction via activation of HIF-1 (Moulin et al., 2022). In turn, ROS and lactate, which are generated with mitochondrial dysfunction, activate mTOR and HIF-1α-related signaling pathways (Tauffenberger et al., 2019; Ip et al., 2017), and the oxidative stress caused by mitochondrial dysfunction may protect the structure and function of HIF-1α by inhibiting its proteasomal degradation (Wu et al., 2023). This shows that there is an interactive relationship between HIF-1 and mitochondrial dysfunction and that the effect of HIF-1 on mitochondria is influenced by the extracellular environment and tissue variability (Figure 3). HIF-1, regulating glycolytic metabolism, can affect the level of protein lactylation by regulating lactate production. The protein lactylation may be one of the effective mechanisms by which HIF-1 regulates HMGBI, a core factor that mediates programmed death in multiple cells. Although the mechanisms of HIF-1-mediated neuronal loss, which is strongly associated with mitochondrial dysfunction-induced epilepsy, have not yet been clarified, it cannot be denied that HIF-1 may regulate the level of HMGBI lactylation to mediate programmed neuronal death or induce mitochondrial dysfunction to mediate the occurrence and development of epilepsy. It is speculated that HIF-1 may be an important target for regulating epilepsy-associated protein lactylation (Figure 6).

### 3.3 Regulation of protein lactylation by HATs and HDACs

#### 3.3.1 Protein lactylation and delactylation catalyzed by HATs and HDACs

Previous studies have suggested that the acetyl group for protein acetylation is mainly supplied by acetyl coenzyme A and that the lactyl group for protein lactylation is mainly supplied by lactate (Dong et al., 2022). Acetyl coenzyme A and lactate in the body are mainly converted from pyruvate, an intermediate product of glycolytic metabolism, and both lactate modification and acetylation modification of proteins can be affected by glycolysis. Furthermore, it has been demonstrated that the mechanism of histone modification by lactate is similar to that of acetyl coenzyme A and that both protein lactylation and protein acetylation are regulated by acetylases and deacetylases (Moreno-Yruela et al., 2022) (Figure 4). Acetyltransferase P300, a newly identified lactonylated writer protein, was shown to promote gene expression by upregulating the levels of H3K9 acetylation, H4K12 acetylation, H4K16 acetylation, H4K12la, and H3K18la at the transcriptional start sites of pluripotency genes (Fan et al., 2023; Lin et al., 2022). P300 belongs to the nuclear A-type HAT. It has been demonstrated that the loss of P300 activity is associated with neuronal survival and long-term memory and that P300 may intervene in neuronal death by catalyzing histone acetylation or lactylation (Lu et al., 2015). P300 promotes PGK1 expression by catalyzing H3K27 acetylation in the PGK1 promoter region. PGK1 exacerbates ischemic stroke-induced brain injury by promoting microglia M1 polarization, inflammation, and glycolysis (Cao et al., 2023). Indeed, the regulation of protein lactylation by P300 can depend not only on the direct catalysis of lactide writing but also on the regulation of lactate production.

HDACs consist of HDAC1-11 and SIRT1-7 with deacetylase activity. A recent study revealed that HDACs, like P300, regulate protein lactylation and acetylation. Class I HDAC1, 2, and 3 are the most efficient lysine delactylases *in vitro*, mainly catalyzing the delactylation of H3K18 and H4K5. However, SIRT1-3, in vitro experiments, were shown to have a slight delactylation activity, and their main regulatory sites are in H3K18 and H3K5 (Fan et al., 2023; Moreno-Yruela et al., 2022). Furthermore, the knockdown of HDAC1-3 resulted in an increase in H4K5la but had little effect on pan-Kla and H3K18la. The differences in histone lactylation sites regulated by HDAC1-3 between in vitro and *in vivo* experiments suggest that the specificity of HDAC action sites may be affected by certain cofactors (Fan et al., 2023). However, some studies have suggested that HDAC1-3 predominantly regulates the level of H3K18la both in vitro and in vivo and that MS-275, a selective inhibitor of HDAC1-3, promotes gene expression by upregulating the levels of H3K14la and H3K18la (Dai et al., 2022). H3K18la can be widely distributed in the cells and tissues of humans and mice. Importantly, it has been discovered that H3K18la not only accumulates at promoters but also enriches in a tissue-specific manner at active enhancers, sharing a genomic distribution pattern with H3K27 acetylation (Galle et al., 2022). Mitochondrial dysfunction can promote pan-lysine lactylation and H3K18 lactylation without affecting pan-lysine acetylation and H3K27 acetylation (He et al., 2023). Lactylation and acetylation of histones may cooperate to execute the transcriptional regulation of genes. The acetylation of histones can competitively inhibit histone lactylation (Rho et al., 2023). However, HDAC inhibitors may activate neuronal transcriptional programs by mediating chemical modification of multiple forms of histone lysines, such as H3K9 acetylation, H3K9 crotonylation, H3K18 crotonylation, and H3K18la. There may be collaborative, rather than competitive, relationships between histone acetylation, butyrylation, and lactic acidification in neuronal cells (Dai et al., 2022). HDACs can not only regulate histone lactylation levels but also regulate lactylation modifications of non-histone proteins. SIRT1 is a potential non-histone delactylase in mammals (Sun et al., 2022). Meanwhile, HDACs affect the level of protein lactylation by regulating glucose metabolism. For example, SIRT1 promotes lactate production by positively regulating the expression of HK-2, a key enzyme in glycolysis (Chen et al., 2022) (Figure 5), while SIRT6 has been shown to be an inhibitor of glycolysis (Sun et al., 2021) (Figure 1). SIRT3 promotes the conversion of pyruvate to acetyl coenzyme A by catalyzing the deacetylation of PDH, thereby reducing lactate production and downregulating the lactylation of mitochondrial proteins (An et al., 2023).

#### 3.3.2 The role of HATs and HDACs in the regulation of HIF-1

HDACs regulate HIF-1α activity through various mechanisms. HDACs can regulate the activity of HIF-1α by affecting its expression level, degradation rate, and post-translational modifications (Table 2). Various factors may influence this process, such as tissue specificity and external environmental pressures (Geng et al., 2011).

**Table 2:** The histone deacetylases regulating transcription factor HIF-1α.

Members	Subjects	Associated diseases	Mechanisms and effects	References
HDAC1	HEK293 T, SW48, and LOVO cell lines	Colorectal cancer	The HDAC1 may activate the HIF1α/vascular endothelial growth factor A signaling pathway by directly inhibiting the ubiquitination of HIF1α, which can promote tumor angiogenesis.	Chen et al. (2020)
	HAEC lines	Atherosclerosis	The HDAC1 reduces ROS accumulation and endothelial cell apoptosis in atheromatous plaques through downregulation of HIF1α expression	Wang et al. (2021)
	PC cell lines	Pancreatic Cancer (PC)	The HDAC1 can improve HIF-1α activity, which can promote aerobic glycolysis in tumor cells by inhibiting acetylation or degradation of HIF-1α.	Jin et al. (2021)
HDAC2	BV-2 microglial cells	Postoperative cognitive dysfunction	The HDAC2 may promote HIF-1α/PFKFB3 axis-mediated neuroinflammatory injury by increasing the expression of HIF-1α.	Liu et al. (2022)
	Human umbilical cord mesenchymal stem cell	Esophageal cancer	Blocking the binding of HDAC2 to the promoter of HIF-1α can activate the transcription of HIF-1α, which can promote tumor growth and metastasis.	Chen et al. (2023)
HDAC4	Osteoblast precursor cells	Diabetic osteoporosis	The HDAC4 can inhibit bone growth by blocking the HIF-1α/vascular endothelial growth factor A pathway.	Zhang et al. (2023)
HDAC5	Hippocampal neurons	Epilepsy	The HDAC5 may promote the synthesis of inflammatory factors IL-1β, TNF-α, and IL-6 by upregulating the expression of HIF-1α and PFKFB3, thereby inducing neuronal cell apoptosis, oxidative stress, and inflammation.	Pan et al. (2021)
	Rat Pheochromocytoma (PC)-12 Cells	Intermittent hypoxia	The HDAC5 can not only downregulate the transcription level of HIF-1 by reducing the acetylation of histone lysine residues but also inhibit the transcriptional activity of HIF-1 by lowering its acetylation levels.	Wang et al. (2021)
HDAC6	Th17 cell	Acute lung allograft rejection	Tubastatin A may downregulate HIF-1α expression levels and activity by specifically inhibiting HDAC6 activity, which can reduce acute allograft rejection.	Zhou et al. (2020)
HDAC7	Mouse macrophages	Inflammatory disease	The HDAC7-u, without regulating the transcriptional level of HIF-1α, may enhance HIF-1α transcriptional activity during hypoxia by binding to it, which can promote the transcription of HIF-1 α target genes.	Shakespear et al. (2013)
HDAC8	Human melanoma cell line A2058	Melanoma	The HDAC8 promotes the expression of HIF-1α target genes, such as hexokinase 2 (HK2) and glucose transporter protein 1 (GLUT1), by upregulating HIF-1α expression levels and elevating HIF-1α transcriptional activity, which can promote the proliferation and metastasis of melanoma cells.	Kim et al. (2023)
HDAC9	Primary cortical neurons	Ischemic stroke	The HDAC9 enhances the transcriptional activity of HIF-1 by catalyzing its deacetylation, thereby mediating ischemic stroke-induced neuronal iron death in the cortical layer.	Sanguigno et al. (2023)
SIRT1	bEnd.3 cells	Ischemic stroke	The Sirt1 may block HIF-1α/NOX2 signaling cascade-mediated microvascular endothelial cell destruction by promoting HIF-1α degradation.	Li et al. (2022)
	Stromal vascular fraction cells	Adipose fibrosis	The SIRT1 can inhibit the transcription of HIF-1 target genes by downregulating the expression level and acetylation level of HIF-1α.	Wu et al. (2023)
	Neuronal cells	Parkinson’s Disease	The SIRT1 can suppress oxidative stress by maintaining HIF-1α in a deacetylated state to exert neuroprotective effects.	Ubaid et al. (2022)
SIRT2	Hippocampal neurons	Ischemic stroke	The SIRT2, in neurons, can downregulate the expression of HIF-1a under hypoxic conditions.	Kaitsuka et al. (2020)
SIRT3	A549 cells	Viral infections	Degradation of SIRT3 in damaged mitochondria increased cellular mROS levels to enhance HIF-1α stability and its target gene expression.	Gong et al. (2021)
	Primary murine juvenile chondrocytes	Osteoarthritis	The loss of Sirt3 may increase the expression of HIF-1a to induce a shift of chondrocyte metabolism from mitochondria to glycolysis.	Zhu et al. (2022)
	Bone marrow-derived macrophages (BMDMs)	Hepatic ischemic/reperfusion injury	The plasma membrane-bound G protein-coupled bile acid receptor knockdown promotes the acetylation, ubiquitination, and degradation of Forkhead Box Protein O3 by inhibiting SIRT3 expression, resulting in increased HIF-1α transcription.	Wang et al. (2020)
SIRT4	786-O cells and Caki-2 cells	Clear cell renal cell carcinomas	The SIRT4 binds to HIF-1α in a protein–protein interaction and directly inhibits HIF-1α expression, thereby blocking HIF-1α/HO-1-mediated tumor cell growth.	Tong et al. (2021)
SIRT6	PC9 cells and HCC827 cells	Non-small cell lung cancer	The SIRT6 may promote the growth of erlotinib-resistant non-small cell lung cancer cells by activating the HIF-1α/HK2 signaling axis to promote aerobic glycolysis in tumor cells.	You et al. (2022)
SIRT7	Hep3B cells	Unknown	Overexpression of Sirt7, without its deacetylation activity, can lead to a significant decrease in HIF-1α protein levels.	Hubbi et al. (2013)

It was shown that HDAC1 and HDAC2 downregulate the expression of fructose-1,6-bisphosphatase 1 by catalyzing the deacetylation of histone H3K27 in the enhancer of 1,6-bisphosphatase. Fructokinase 1,6-bisphosphate, a rate-limiting enzyme in gluconeogenesis, reduces the onset of glycolysis and decreases lactate production by inhibiting the expression of HIF-1α, thereby inhibiting histone lactylation (Yang et al., 2017; Fan et al., 2020). Meanwhile, HDAC2 promotes HIF-1α/6-phosphofructo-2-kinase (PFKFB3) axis-mediated neuroinflammatory injury by increasing the expression of HIF-1α (Liu et al., 2022). However, not all HDACs can act as upstream regulators of HIF-1, and there are currently few reports on the regulation of HIF-1 function by HDAC3, HDAC10, HDAC11, and SIRT5. It has even been suggested that HIF-1α may promote HDAC3 expression, serving as an upstream regulator of HDAC3 (Wang et al., 2021) (Figure 5). However, in epileptic hippocampal neurons, HDAC5 promotes HIF-1α enrichment at the PFKFB3 promoter by upregulating HIF-1α expression, thereby contributing to PFKFB3-induced neuronal apoptosis, oxidative stress, and inflammation (Pan et al., 2021).

Under hypoxic conditions, HDAC7-u enhances the transcriptional activity of HIF-1α, promoting the transcription of HIF-1α target genes. This effect is achieved by increasing HIF-1α’s transcriptional activity rather than altering its transcriptional level (Shakespear et al., 2013). HDAC9 enhances the transcriptional activity of HIF-1 by catalyzing HIF-1 deacetylation, thereby mediating ischemic stroke-induced neuronal Ferroptosis in cortical layers (Sanguigno et al., 2023).

SIRT1 stabilizes Von Hippel-Lindau activity by deacetylating Von Hippel-Lindau, effectively reversing its acetylation. This modification enhances the stability and function of the Von Hippel-Lindau protein. Under normoxic conditions, hydroxylated HIF-1α accelerates HIF-1α degradation by binding to von Hippel–Lindau ubiquitin ligase complexes (Li et al., 2022; Sailhamer et al., 2010). Activation of HIF-1α under hypoxic conditions induces a shift in energy metabolism from oxidative phosphorylation to glycolysis, which facilitates cellular adaptation to the hypoxic environment to protect neuronal viability (Kaitsuka et al., 2020; Zhu et al., 2022). The knockdown or inhibition of the activities of SIRT2 and SIRT3 has been shown to increase the expression of HIF-1α, which in turn activates cellular glycolysis to ensure the energy supply for neurons in the ischemic core (Kaitsuka et al., 2020; Zhu et al., 2022). The glycolysis in neurons depletes NAD^+^, which leads to the loss of biological activity of the NAD^+^-dependent deacetylase SIRT1, thereby maintaining the stability of HIF-1α structure and function (Li et al., 2022). Blocking the negative regulatory effect of HDACs on HIF-1α activity may help brain tissue adapt to the hypoxic environment to a certain extent, but elevated HIF-1α activity may increase the risk of inflammatory injury (Li et al., 2022; Kaitsuka et al., 2020; Zhu et al., 2022). Furthermore, SIRT6 independently upregulated HIF-1α expression to promote glycolysis (You et al., 2022).

It was shown that P300 may intervene in the functioning of the central nervous system by regulating the activity of key enzymes of glycolysis (Cao et al., 2023; Lu et al., 2015) and that P300/CBP may enhance the expression of HIF-1α target genes by promoting lysine acetylation of the transcription factor HIF-1α (Wang et al., 2024). It can be speculated that P300 may indirectly regulate protein lactylation modification by affecting the lactate production of glycolysis by modulating the transcriptional activity of HIF-1α.

In summary, HAT and HDAC regulate protein lactylation *in vivo*. This regulation depends not only on the catalytic activities of these enzymes but also on the availability of the lactate donor, lactide. These enzymes also indirectly regulate the expression of proteins related to glycolytic metabolism, which are induced by the transcription factor HIF-1α. Additionally, HAT and HDAC directly influence the activity of key glycolytic enzymes, further impacting lactate production. These mechanisms highlight the complex interplay between histone modification and metabolic control in cellular processes.

### 3.4 Regulation of HIF-1 by other substances

Research indicates that the stability and activation of HIF-1 are not only regulated by HATs and HDACs but also closely related to oxygen levels, cellular iron content, and the activation of other signaling pathways (Liang et al., 2023).

The autophagy-related factor P62 may enhance the expression of glycolysis-related proteins by upregulating HIF-1α expression (Calvo-Garrido et al., 2019). In turn, the products and key enzymes of glucose metabolism can adjust the biological activity and expression level of HIF-1. In microglia, lactate not only upregulates the transcriptional level of HIF-1α by promoting the histone H4K12la of the HIF-1α promoter (Pan et al., 2022; Jiang et al., 2021) but also enhances the production of lactate by directly promoting the lactylation of HIF-1α (Luo et al., 2022).

Accumulating lactate may promote the expression of the transcription factor STAT6 by upregulating histone lactylation in the STAT6 promoter region (Lu et al., 2022). Furthermore, STAT6 may reduce HIF-1α expression by inhibiting the mTOR-mediated glycolipid metabolism regulation signaling pathway (Lu et al., 2022; Park et al., 2019) (Figure 3).

Meanwhile, PDP1 can drive the acetylation of histone H3 in the promoter of HIF-1 target genes and promote gene expression by promoting the production of the glucose metabolism intermediate product acetyl coenzyme A. The expression of the HIF-1 target gene PDK1 can counteract the effects of PDP1 and diminish the biological impact of HIF-1 (Karagiota et al., 2023) (Figure 5). The epigenetically important regulators HAT and HDAC have been shown to be major regulators of lactylation modification, and a large number of studies have demonstrated that HAT and HDAC are critical for the progression of epilepsy (Wang et al., 2022). The regulation of HIF-1 by HAT and HDAC may be a potential pathway for their intervention in epilepsy-associated protein lactylation.

## 4 The impact of HDAC inhibitors on lactylation and epilepsy

Although the state of protein acetylation depends on the balance between HAT and HDAC, previous studies have shown that the imbalance between HAT and HDAC favors the latter, so the clinical development of HDAC inhibitors is focused on restoring this balance (Bezu et al., 2019). Currently, some HDAC inhibitors have been applied and studied as antiepileptic drugs (Wang et al., 2022). The protein lactylation induced by lactate is regulated by HAT and HDAC as protein acetylation (Yang et al., 2023). Suberoylanilide hydroxamic acid and trichostatin A, which are HDAC inhibitors, may reduce the rate of lactate production from glycolysis by promoting acetylation of key enzymes of glycolytic metabolism, thereby reducing protein lactylation modification (Wu et al., 2023; Zhang et al., 2022). It is undeniable that the antiepileptic effect of HDAC inhibitors may be partially mediated by the modulation of protein lactylation modifications.

### 4.1 The impact of HDAC inhibitors on neuronal loss and glial activation

The common HDAC inhibitors can be divided into four groups based on their structure: butyric acid derivatives (e.g., valproic acid), hydroxamic acid derivatives (e.g., suberoylanilide hydroxamic acid), benzamides (e.g., entestat) and cyclic tetrapeptides (e.g., romidepsin) (Bezu et al., 2019). Sodium butyrate can alleviate neurological damage caused by cerebral hypoxia-ischemia. The ability of trichostatin A to stimulate neurogenesis in the subgranular zone of the hippocampus may facilitate recovery from hypoxic–ischemic injury in the neonatal brain (Zalewska et al., 2020).

The conventional antiepileptic drug valproate may inhibit the activation of the NF-κb signaling pathway by promoting the acetylation of STAT1 and p65, thereby impeding microglia activation and attenuating I/R-induced neuroinflammation and brain damage (Dai et al., 2021). Suberoylanilide hydroxamic acid has been shown to prevent astrocyte and microglia activation, which can alleviate ischemia-induced neuroinflammation by inhibiting the deacetylation activity of HDAC1/2 (Dai et al., 2021), while the class IIa HDAC inhibitor MC1568 attenuates ischemic stroke-induced ferroptosis of cortical neurons by blocking HDAC9 transcription (Sanguigno et al., 2023). Furthermore, HDAC inhibitors significantly inhibit the immunological activation and aggregation induced by cerebral hemorrhage. Scriptaid has been proven to alleviate the inflammatory injury of cortical neurons caused by traumatic brain injury or cerebral hemorrhage by promoting the polarization of microglia/macrophages toward the protective M2 phenotype (Dai et al., 2021). Cerebral hemorrhage is highly susceptible to epilepsy, and the class I HDAC inhibitor entinostat protects neurons by inhibiting microglia activation, thereby ameliorating cerebral hemorrhage-induced neuroinflammatory damage (Bonsack and Sukumari-Ramesh, 2021). The class I HDAC inhibitor CI-994 and the HDAC1-specific inhibitor parthenolide may enhance synaptic plasticity in hippocampal neurons by inhibiting astrocyte and microglia immunoreactivity, thereby ameliorating epilepsy-induced cognitive impairment (Jia et al., 2023; Dai et al., 2021; Burns et al., 2022). RGFP966, the HDAC8-specific inhibitor WK2-16, and the HDAC3-specific inhibitor BG45 have been shown to reduce neuroinflammation-mediated hippocampal neuronal loss by inhibiting the proliferative activation of glial cells, thereby maintaining the morphological and functional stability of the cerebral cortex (Dai et al., 2021; Wang et al., 2023). Additionally, FK228, also known as romidepsin, can exert a neuroprotective effect by inhibiting NETosis-mediated neural inflammation and promoting the regeneration of neurons (Thakur et al., 2024). HDAC inhibitors may reduce the occurrence of post-stroke epilepsy by alleviating neuronal loss and neuroinflammation induced by ischemic brain injury.

### 4.2 The biological activity of HIF-1 is regulated by HDAC inhibitors

HIF-1α not only affects ischemia-induced hippocampal neuronal loss by regulating lactate metabolism but is also closely associated with epilepsy-related hippocampal neuronal death and oxidative stress (Liang et al., 2023; Zhao et al., 2022). Valproate, which has been shown to inhibit class I and class II HDACs, may reduce HIF-1α-mediated hippocampal neuronal loss by lowering the protein level of HIF-1α, thereby achieving antiepileptic effects (Luo et al., 2013; Simeone et al., 2017). Sodium butyrate not only enhances ubiquitination of HIF-1α Lys532 by catalyzing its acetylation to target HIF-1α for proteasomal degradation (Naia et al., 2017), but also upregulates the expression level of HIF-1α by inhibiting HDAC2 (Bashir and Olaniyi, 2023). Moreover, butyrate sodium combined with curcumin, which is a less toxic natural pan-HDAC inhibitor derived from food, prevents PI3K/Akt axis-mediated inhibition of HIF-1α activity through inhibition of HDAC1 (Islam et al., 2023). The development of food-borne HDAC inhibitors may be beneficial for epilepsy control. The ketogenic diet can increase the production of β-hydroxybutyrate, which may alleviate seizures associated with neuroinflammation by promoting the acetylation of histone H3K9 and H3K14, similar to HDAC inhibitors (Simeone et al., 2017).

Suberoylanilide hydroxamic acid, vorinostat, which is the first acetylation-modifying drug approved by the US Food and Drug Administration, mainly inhibits the activity of HDAC1 and HDAC2. It has been demonstrated that vorinostat inhibits the activity of HDACs, including HDAC4, to promote HIF-1α acetylation, which can inhibit the biological activity of HIF-1α and promote HIF-1α degradation (Geng et al., 2011; Sailhamer et al., 2010). Meanwhile, vorinostat promotes intracellular lactate efflux by upregulating the expression of MCT1 and MCT4, and lactate released into the extracellular space enhances the inhibitory effect of vorinostat on HDAC activity (Radoul et al., 2019). In addition, N-hydroxy-7-(2-naphthylthio) heptanamide, a novel synthetic HDAC inhibitor, was shown to inhibit HIF-1α expression to a greater extent than Vorinostat by *in vitro* and in vivo experiments in breast cancer (Park et al., 2011). Panobinostat (LBH589), a novel pan-HDAC inhibitor, has been shown to potently inhibit the viability of HIF-1α (Ni and Ni, 2021). The pan-histone deacetylase inhibitor PCI-24781 induced cellular autophagy by upregulating the concentration of HIF-1α (Bhalla et al., 2013).

In summary, as HIF-1α can affect the level of protein lactylation by regulating the expression of key enzymes of glycolysis and lactate transporter proteins, HDAC inhibitors may control ischemic stroke-induced protein lactylation by regulating the activity of HIF-1α, thereby alleviating abnormal excitation of neurons (Hagihara et al., 2021; Wu et al., 2023; Zhang et al., 2022). It may be one of the potential mechanisms by which HDAC inhibitors could help prevent post-ischemic stroke epilepsy.

## 5 Discussion

Stroke is one of the leading causes of death worldwide. Ischemic strokes account for 87% of strokes in humans (González-Rodríguez and Fernández-López, 2023). The accumulation of lactate caused by ischemic stroke may mediate neuroexcitotoxicity and neuronal apoptosis through the upregulation of protein lactylation levels, which can lead to post-stroke epilepsy (Hagihara et al., 2021; Wang et al., 2022; Zhang et al., 2023). Reperfusion injury in ischemic foci of the brain is mainly mediated by mitochondrial dysfunction, which can be induced through over-activated glycolysis (She et al., 2023; Liu et al., 2023). Although reperfusion of blood improves the oxygen supply to the ischemic lesion, it does not ensure that the mode of energy metabolism in the ischemic lesion and its surrounding tissues is switched from glycolysis to oxidative phosphorylation, implying that reperfusion may not completely block lactate production. Although there is no evidence that neurons initiate the “Warburg effect,” also known as “aerobic glycolysis,” as tumor cells do (Lin et al., 2022), peripheral immune cells differentiated from bone marrow hematopoietic stem cells have been observed to display the “Warburg effect,” such as macrophages and neutrophils (Chen et al., 2022). The “Warburg effect” of tumor cells is primarily for the rapid proliferation of tumor cells (Lin et al., 2022), while the “Warburg effect” of peripheral immune cells promotes an inflammatory cascade by supporting the expression of pro-inflammatory cytokines during the activation of immune cells (Zhang et al., 2019). Brain injury typically involves the disruption of the blood–brain barrier, and peripheral blood immune cells can easily migrate across the damaged barrier into the brain, where they work together with activated glial cells to exacerbate neuroinflammatory damage (Li et al., 2021; Bernis et al., 2023). After the restoration of blood supply to brain tissue, peripheral immune cells may continue to produce lactate and release it extracellularly, relying on the Warburg effect, and the lactate may be taken up by neurons via the MCT to promote neuronal protein lactylation, which may increase the risk of post-stroke epilepsy by mediating neuronal excitation (Hagihara et al., 2021; Zhang et al., 2019; Mayorga-Weber et al., 2022; Tröscher et al., 2021).

Furthermore, the enhancement of neuronal excitability further upregulates protein lactylation levels by promoting the conversion of glucose to lactate in the brain (Li and Freeman, 2015). However, as the lactate from aerobic glycolysis increases in peripheral immune cells infiltrating brain tissue, the level of protein lactylation is significantly upregulated, which can induce a shift of activated peripheral immune cells to an anti-inflammatory state for avoiding an unlimited extent of inflammatory damage to brain tissue (Zhang et al., 2019). It is evident that protein lactylation modifications mediate different biological effects in different cells.

Although protein lactylation alleviates ischemic stroke-induced neuroinflammation by affecting the activity of peripheral immune cells migrating to the ischemic lesion, the protein lactylation-mediated neuronal loss and glial cell activation should not be underestimated (Pan et al., 2022; Barros et al., 2023; Yang et al., 2024; Wu et al., 2023; Yao et al., 2023). The modification of protein lactylation in neurons and glia is associated not only with the facilitation of glycolysis but also with the cellular transport of lactate. If both lactate production and intracellular transformation of lactate in neurons can be controlled, post-stroke epilepsy would be better prevented. Since HIF-1 can act as a transcription factor for key enzymes of the glycolytic pathway and lactate transporter proteins, inhibiting the transcriptional activity of HIF-1 may be a potential measure to combat post-stroke epilepsy (Longhitano et al., 2022; Karagiota et al., 2023; Huang et al., 2022). HDAC may affect the expression of HIF-1-related target genes by directly or indirectly regulating HIF-1 transcription, degradation, and bioactivity, thereby influencing cellular glucose uptake, mitochondrial function, and lactate metabolism (Table 2). However, research has confirmed that lactate increases the activity of SIRT1, which is a potential protein lactylation and acetylation modification regulator in mammals (Sun et al., 2022; He et al., 2023), and the ketogenic diet may exert an anti-epileptic effect by inhibiting hypoxia-enhanced glycolysis (Sun et al., 2021; Hou et al., 2017). Thus, inhibition of lactate production and lactate-mediated protein lactylation may be the key to reducing post-stroke epilepsy rather than inhibition of HDAC enzyme activity, and it has been demonstrated that the HDAC inhibitors sodium butyrate and trichostatin A control lactate production by modulating the activities of pyruvate kinase and LDH (Rodrigues et al., 2015).

HMGB1 is a core factor mediating programmed cell death in various cells, and the nucleus-to-cytoplasm translocation of HMGB1 in neurons and glial cells may be an important trigger for status epilepticus (Pauletti et al., 2019; Chen et al., 2023; Tang et al., 2023). It has been demonstrated that N-(2′-hydroxyphenyl)-2-propylvaleramide may promote HMGB1 acetylation through inhibition of HDAC1, thereby inducing translocation of HMGB1 from the nucleus to the cytoplasm. Meanwhile, N-(2′-hydroxyphenyl)-2-propylvaleramide also induced HMGB1 secretion by promoting ROS synthesis (Sixto-López et al., 2020). The HDAC8-specific inhibitors, such as PCI-34051 and PCI-48012, and Scriptaid, all promote HMGB1 acetylation, which triggers HMGB1-mediated biological effects by inhibiting HDAC activity (Lopez et al., 2015; Chi et al., 2017). However, the mechanism of HDAC inhibitors regulating the biological effects of HMGB1 includes promoting the HMGB1 acetylation and regulating HMGB1 expression. Chidamide, a novel oral selective HDAC inhibitor, can downregulate the expression of HMGB1 (Liu et al., 2021). The novel HDAC inhibitor HFY-4A induces apoptosis by upregulating HMGB1 expression (Yin et al., 2023). The HDAC inhibitor pemetrexed, combined with sildenafil, may upregulate the expression of HMGB1 and promote the extracellular release of HMGB1 by inhibiting HDAC6, HDAC2, HDAC4, and HDAC9 (Booth et al., 2017). In addition, HDAC inhibitors promote the formation of the HMGB1-P53 complex by inhibiting SIRT1 activity, thereby reducing lactate generated from glycolysis (He et al., 2023; Li et al., 2023; Ma et al., 2020; Wang et al., 2023). HDAC inhibitors may regulate the activity of HMGB1 in various ways, thereby indirectly modulating protein lactylation and cell death.

HDAC can affect lactate production by regulating the activity of the transcription factor HIF-1 or key enzymes of glycolysis. Conversely, lactate can inhibit HDAC activity like conventional HDAC inhibitors (Figure 5). Lactate has been shown to induce histone H3 and H4 hyperacetylation, which may promote the expression of some genes by inhibiting class I and II HDAC (Wagner et al., 2015). In CD8^+^ T cells, lactate promotes protein acetylation by inhibiting HDAC activity, thereby inhibiting apoptosis (Feng et al., 2022). Lactate promotes the immunosuppressive effect of tumors by blocking the formation of a transcriptional repression complex between NF-κB and HDAC3 (Chang et al., 2021). Lactate can enhance HDAC6 activity by inhibiting HDAC11 activity, and HDAC6 upregulates IL-10 transcript levels and blocks the inflammatory effects of immune cells by promoting histone H3 acetylation in myeloid-derived suppressor cells and macrophages (Heim et al., 2020). Existing studies have confirmed that lactate can exert neuroprotective effects by regulating the expression levels of HDAC2/3/5 (Ding et al., 2020; Puri et al., 2019; Karnib et al., 2019; Genders et al., 2019). Lactate can alleviate inflammatory damage and protect neurological functions by modulating the function of HDACs.

There is a feedback regulation of HDAC expression by HIF-1α. HIF-1α in a hypoxic environment can enhance the deacetylation activity of HDAC2 (Wang et al., 2022). Meanwhile, HIF-1α can downregulate the transcript level of HDAC4 by binding to the promoter of HDAC4 (Pan and Zhao, 2021). The hypoxic environment drives HIF-1α to bind directly to the HDAC3 promoter, which reduces histone acetylation by upregulating HDAC3 expression (Wang et al., 2021; Yuan et al., 2022). However, the regulatory effects of HIF-1α on HDACs, like lactate, were not consistent. It has been shown that HIF-1α impairs the anti-inflammatory effect of Treg cells on pro-inflammatory macrophages by promoting the expression of SIRT2 in Treg cells, thereby contributing to ischemia- and hypoxia-induced neuroinflammatory injury (Shu et al., 2019). Both lactate and HIF-1α can act as HDAC inhibitors to regulate protein acetylation modifications. Based on the report of lactate mediating protein phosphorylation (Maschari et al., 2022), it is speculated that lactate not only induces protein lactylation but also alters protein function by regulating the levels of protein phosphorylation and acetylation.

Recent studies have suggested that histone acetylation and lactonylation may compete (Rho et al., 2023; Moreno-Yruela et al., 2022). The competition between the lactoyl and the acetyl groups for the epigenetic modifications of histone lysine is related not only to the activity of HDAC but also to the concentrations of lactic acid and acetyl coenzyme A. Pyruvate can be converted to lactate or acetyl coenzyme A by different enzymes, and the type of histone modification can be altered by regulating the activity of these enzymes (Dai et al., 2022). It has been shown that proteins tend to undergo acetylation rather than lactylation in the physiological state, while in hypoxia, histone lactylation can replace histone acetylation to regulate cellular function (Dai et al., 2022). However, it remains to be further explored whether ischemic stroke can mediate protein lactylation, replacing protein acetylation, which may alter cellular function by promoting glycolysis. Revealing this competitive relationship between lactylation and acetylation may facilitate the design of new therapeutic strategies for epilepsy after ischemic stroke. In addition, lactate and its mediated modification of protein lactylation can affect neuronal and glial cell function and the functional recovery of ischemic foci by modulating vascular endothelial cell function (Fan et al., 2023).

In summary, the modulation of protein lactylation levels by regulating lactate production and/or lactate membrane transport may be a potential strategy to combat epilepsy after ischemic stroke.

## 6 Conclusion

Ischemic brain injury results in lactate accumulation within the central nervous system, contributing to metabolic dysregulation. Lactate may induce post-stroke epilepsy by promoting protein lactylation in brain tissue. HAT and HDAC have been shown to modulate cellular function by catalyzing protein lactylation and delactylation, thereby mediating neuronal loss and glial cell activation, which are important factors contributing to epilepsy-associated neuronal hyperexcitability. Although the specific mechanism through which protein lactylation influences post-stroke epilepsy has not yet been validated, conducting research in this area may provide a new theoretical basis for using HDAC inhibitors in the clinical prevention and treatment of post-stroke epilepsy.
